# WImpiBLAST: Web Interface for mpiBLAST to Help Biologists Perform Large-Scale Annotation Using High Performance Computing

**DOI:** 10.1371/journal.pone.0101144

**Published:** 2014-06-30

**Authors:** Parichit Sharma, Shrikant S. Mantri

**Affiliations:** 1 Centre for Development of Advanced Computing (C-DAC), Pune, India; 2 National Agri-Food Biotechnology Institute (NABI), Mohali, India; University of Cyprus, Cyprus

## Abstract

The function of a newly sequenced gene can be discovered by determining its sequence homology with known proteins. BLAST is the most extensively used sequence analysis program for sequence similarity search in large databases of sequences. With the advent of next generation sequencing technologies it has now become possible to study genes and their expression at a genome-wide scale through RNA-seq and metagenome sequencing experiments. Functional annotation of all the genes is done by sequence similarity search against multiple protein databases. This annotation task is computationally very intensive and can take days to obtain complete results. The program mpiBLAST, an open-source parallelization of BLAST that achieves superlinear speedup, can be used to accelerate large-scale annotation by using supercomputers and high performance computing (HPC) clusters. Although many parallel bioinformatics applications using the Message Passing Interface (MPI) are available in the public domain, researchers are reluctant to use them due to lack of expertise in the Linux command line and relevant programming experience. With these limitations, it becomes difficult for biologists to use mpiBLAST for accelerating annotation. No web interface is available in the open-source domain for mpiBLAST. We have developed WImpiBLAST, a user-friendly open-source web interface for parallel BLAST searches. It is implemented in Struts 1.3 using a Java backbone and runs atop the open-source Apache Tomcat Server. WImpiBLAST supports script creation and job submission features and also provides a robust job management interface for system administrators. It combines script creation and modification features with job monitoring and management through the Torque resource manager on a Linux-based HPC cluster. Use case information highlights the acceleration of annotation analysis achieved by using WImpiBLAST. Here, we describe the WImpiBLAST web interface features and architecture, explain design decisions, describe workflows and provide a detailed analysis.

## Introduction

The function of a newly sequenced gene can be discovered by determining its sequence homology with a known protein or family of proteins. The Basic Local Alignment Search Tool (BLAST) is the most extensively used sequence analysis program for sequence similarity search in large databases of sequences [1]. The chances of determining the function of new sequences are increasing every day with the continual unprecedented growth in size of DNA and amino acid databases. BLAST uses a heuristic algorithm and was designed to overcome the impractical nature of dynamic programming algorithms for searching large databases without the use of supercomputers and other specialized hardware [2], [3].

The National Center for Biotechnology Information (NCBI) maintains the public interface of BLAST (http://www.ncbi.nlm.nih.gov/blast), and keeps improving it by adding new features. The NCBI BLAST portal is routinely used by biologists for doing a sequence similarity search for their genes of interest. With the advent of next generation sequencing (NGS) technologies it has now become possible to study gene expression at a genome-wide scale through RNA-seq and metagenome sequencing experiments. Functional annotation of the genes is done by sequence similarity search against multiple protein databases. This annotation task is computationally very intensive if done on standalone desktop or server machines, and will take days to obtain complete results.

The program mpiBLAST is an open-source parallelization of BLAST that achieves superlinear speedup [4]. It was developed to divide and distribute BLAST searches across multiple nodes and multiple processors to obtain results faster. It has been extensively used to accelerate research at many universities, institutes and hospitals (http://www.mpiblast.org). The optimized implementation of mpiBLAST has shown linear scaling on 32768 cores on the Blue Gene/P supercomputer [5].

There is a steep learning curve for biologists to gain the programming skills and expertise in the command line syntax necessary to use high performance computing (HPC) clusters, and although many parallel bioinformatics applications are available in the public domain, researchers are reluctant to use them for this reason. Without specialized training and independent study, it is difficult for biologists to understand the application-specific terminologies and supercomputer architecture. With these limitations it becomes difficult for biologists to use mpiBLAST for accelerating annotation. On the other hand, web servers are more popular amongst biologists as it is not necessary for them to install such useful tools themselves. Until now, a web interface for mpiBLAST has not been available in the open-source domain. Our main objective was to develop a web interface to facilitate the use of HPC clusters by biologists.

We present here the WImpiBLAST portal that we have developed to help biologists to overcome this limitation by having them use a high performance computing cluster for computationally intensive annotation jobs through a simple web interface. WImpiBLAST is a user-friendly and powerful open-source web interface for parallel BLAST searches. The following sections discuss in detail the planned features, design decisions, architecture, workflows, implementation and use case studies in the development of WImpiBLAST.

## Design

### 1. Feature-centric analysis of existing web portals catering for BLAST searches

We analyzed the characteristics of some of the most widely used and feature-rich web portals currently available for bioinformatics applications in order to arrive at the most practical combination of features that should be included in WImpiBLAST. We have summarized our comparison of different web portals and their respective features in Table 1.

**Table 1:** Comparative analysis of existing bioinformatics web portals catering for BLAST search.

Characteristics	Web portals				
	NCBI BLAST WebInterface	wwwblast WebInterface	SequenceserverWeb Interface	PoPLAR ScienceGatewayWeb Interface	Yabi Web Interface
Ease of Installation	Not Applicable	Difficult	Easy	Data Unavailable	Data Unavailable
Robustness(fault tolerance and reporting)	Yes	No	No	Data Unavailable	Yes
Accessibility from multipleplatforms	Yes(web browserbased access)	Yes (webbrowser based)	Yes (webbrowser based)	Yes (webbrowser based)	Yes (webbrowser based)
Logging functionality	Yes	No	No	Yes	Yes
Multi-threadedexecution	Yes	Yes	Yes	DataUnavailable	Yes
Multi-nodeexecution	Yes	No	No	Yes	DataUnavailable
Suitability for largescale annotation	No	No	No	Yes	DataUnavailable
Source Code availability(Open Source)	No	Yes	Yes	No	Yes

For our study, we selected NCBI’s BLAST web interface [6], wwwblast [7], Sequence Server [8], the PoPLAR science gateway [9] and the Yabi web interface [10] in order to establish common guidelines for web portal design and development. Since these interfaces vary greatly in terms of the features they support, we focused on the following characteristics to compare these web portals:

- Ease of installation (time and effort)
- Robustness and fault tolerance
- Accessibility from multiple platforms
- Support for logging and result saving
- Support for multi-threaded or multi-node execution.

After analyzing the above mentioned bioinformatics portals and their relevant features, we concluded that the characteristics mentioned in Table 1 are essential for a scientific portal to ensure user satisfaction and acceptance by a wide audience of both technically experienced and novice users. An in-depth comparison of the above mentioned bioinformatics portals and command line tools, including technical aspects, are given in Tables 2 and 3. This study helped us to determine the feasible trade-off between user-satisfaction-led design decisions and the development effort required to implement them.

**Table 2:** Feature oriented comparison of command line open source NCBI BLAST+ and mpiBLAST application.

Features	Applications	
	mpiBLAST (parallel version of NCBI BLAST)	BLAST+
Command line execution	Yes	Yes
Open Source GUI available	No	Yes
Scalable across multiple compute nodes	Yes	No
Error reporting by application	Yes	Yes

**Table 3:** Feature oriented comparison of WImpiBLAST interface with other BLAST search supporting web interfaces.

Features	Sequenceserver(Interfacefor NCBIBLAST)	PoPLAR	Yabi	WImpiBLAST
mpiBLAST integration	No	No	No	Yes
Creation/modification of the job	Yes	Yes	Yes	Yes
Creation/modification of workflows	No	No	Yes	No
Run time job tracking	No	No	DataUnavailable	Yes
Parameter customization	Yes	Yes	Yes	Yes
File upload/download/view	No	Yes	Yes	Yes
Email notifications	No	Yes	Yes	Yes
User authentication	No	Yes	Yes	Yes
Logging or Record keeping	No	Yes	Yes	Yes
Job Administration GUI	No	DataUnavailable	Yes	Yes

### 2. WImpiBLAST: Challenges, Design and Decisions

Web portals can provide single-window access to diverse hardware and software resources and scientific applications in a coherent, easy to use and intuitive manner for the end user, along with cross-platform accessibility. Recently, web portals and standalone web interfaces have popularized otherwise complicated command line applications across a huge user base, as evident from the available interfaces for different bioinformatics applications, viz. [6]–[12].

Before initiating development of the WImpiBLAST portal we also analyzed alternative approaches to developing this interface, such as a desktop-based client–server model, developing an interface using a thin client that can interact with remote servers, or a web-based client–server model. However, there were obvious factors that suggested the web-based scenario as a more practical choice for the development of the application interface [13]. Some of the primary benefits are as follows:

- Cross-platform accessibility, as only a web browser is needed to access the web portal.
- Flexibility to update or change the source code, since web interface will be hosted on a central server and new changes will be reflected automatically each time the previous release is replaced by a new one.
- Smooth user experience and minimum load, as no additional installation is required on the user’s system.

Although the web-based approach appeared promising, there were still some critical design decisions that affected the design and development of WImpiBLAST; these are addressed in the following sections.

#### 2.1. Level of abstraction to user

As discussed by Letondal et al. [13] and Untergasser et al. [14], the level of interface abstraction is critical in deciding the comfort level of a user with an interface. We tried to design a simple and feature-rich interface by addressing the following questions:

- Should an interface contain a number of parameters, all of which the user should specify, thus decreasing the level of abstraction at the cost of fine-grained control over the run-time execution of the application, or
- Should an interface only consist of a few, necessary, parameters, and handle most of the application and computational-resource-specific details on its own?

Option (i) would have resulted in a lower development curve (less development time and effort) and reduced code length at the cost of a difficult user experience, as most parameters would be filled in by the user (requiring more effort from them) and hence there would be less validation in the code. However, this strategy had one serious flaw in that it does not account for variation in the level of user exposure to parallel applications and resource management. Therefore, low levels of abstraction may prevent less-experienced users or novices from using the web interface.

Option (ii) would have led to a higher level of abstraction and user friendliness but would also result in a higher development curve, as most parameters would be handled by the code. This strategy promised an easy user experience at the expense of higher development efforts. However, for the sake of experienced users, the interface should also provide the facility to fine-tune the parameters; otherwise, the default settings should take priority.

We decided to implement option (ii), thus letting users easily complete forms as well as change parameter values whenever desired, thereby catering to both advanced and novice users.

#### 2.2. Logging and result saving

The issue of logging and result saving is critical but is not discussed in detail in the literature. There is some description of saving final or intermediate results in [10], [15], but not with respect to error handling or troubleshooting. In the context of web portals, logging can help in terms of recording usage data and resource-specific errors, leading to better administration of the portal.

Since web portals should also cater to the needs of the system administrator by reporting job history, errors and warnings for troubleshooting and record keeping, we tried evaluating the answers to the following questions in order to design robust administration modules:

- Should new models or mechanisms be developed for implementing the logging of usage, errors, warnings and data at the cost of additions to software code, iterative testing and troubleshooting, or
- Should advantage be taken of the default logging features of different software components, logically integrating them to facilitate reporting on the web interface?

Option (i) would have ensured a greater degree of customization over the logging mechanism and fine-grained control over every aspect of the different system components involved in logging, such as the resource manager, job scheduler, compute nodes, and so forth. Initially, this approach appears promising, but it does not ensure reliability and efficiency as new models have to be exhaustively tested before they can be put to use in practice.

Option (ii) satisfied the requirements of efficiency by leveraging the logging mechanisms of the different software components in the WImpiBLAST architecture, for example the logging functionality provided by the resource manager. To implement this option the only requirement would be to integrate the logging mechanisms of the components (resource manager, application logs, etc.) to facilitate reporting in the web interface. Also, since such components (resource manager, web server, etc.) have been thoroughly tested and improved several times, there was little need for additional testing.

We decided to follow the integration approach by implementing option (ii), thereby ensuring the reliability and efficiency of the administration modules.

Although there were other factors that affected the design and development, for the sake of simplicity we have discussed only those factors that directly affect the user and administrator experience.

## Methods

### 1. Architecture

Choosing an architecture to complement the features of WImpiBLAST was a difficult decision, in part because our portal was different from existing web interfaces as it was intended to support parallel sequence searches and job administration by utilizing pre-existing software components (e.g. resource managers, job schedulers, or logging daemons) and be used as a standalone interface [8]. The open-source web interfaces that we studied did not support parallel sequence searches along with job monitoring and management. The motivation to adopt a three-tier architecture for WImpiBLAST also derives from the fact that administrators should be able to install and configure the web portal as a separate component on top of an HPC cluster without drastically changing the software or hardware configurations, while users can use it without having to learn the details of the application.

**Figure 1:**
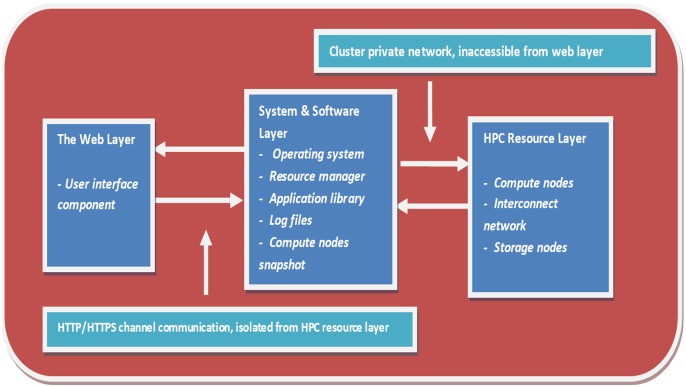
WImpiBLAST three-tier architecture defining non overlapping boundaries between fundamental layers of HPC resources, system software and web layer for efficient bug tracking, layer specific statistical logging and record keeping.

To establish a separation between the different components (the web interface component, system software components and computational resources components), a conceptual three-tiered architecture for WImpiBLAST was adopted as shown in Figure 1. The planned architecture uses design features analogous to CHReME [16] that exploit system, software and resource managers for job control and parallel application execution. However, WImpiBLAST is unique as it provides exclusive job filtering, user-specific filtering and job trace features to administrator. WImpiBLAST is organized as a group of three fundamental layers that includes both software and hardware aspects of the HPC cluster. The primary benefit of isolated layers for partitioning hardware, software and web components is that normal users can be restricted to just the publicly open part of the system, whereas the administrator can make changes to the underlying system components without interfering with any of the users’ data. As the target audience of the portal will only be accessing the web layer, users need not worry about the intricacies of the underlying hardware and software details, formulation of various application-specific or resource management syntaxes, and so on. System administrators can install new applications or update older applications without interfering with the web layer components.

#### 1.1. Layer-specific details: Function and scope

In this section we discuss the primary functionality of each of the layers involved in the three-tier architecture of WImpiBLAST. Also, we will briefly describe the scope of each layer and the isolation between them. In the current implementation each conceptual layer and their components are invisible to the other layers. The web layer interacts with the system layer through a web server, which is a system layer component. Likewise, the system layer interacts with the resource layer through application-specific configuration files that control details of the different computational and data resources. Therefore, the system layer acts as an interface between the web layer and the resource layer while maintaining its own isolation and initiating communication through system and application specific protocols.

**1.1.1. Layer 1: The web layer.** This is responsible for receiving the application-specific, job-specific and file-handling-related inputs from users and transferring them to the system layer after formulating the correct syntax for interpretation by the system software, application runtime and resource manager. Data entered by the user from the web interface is logically incoherent, and hence it will not form any meaningful query until it is organized in a predefined syntactical format that can be interpreted by the application environment and job scheduler. System layer components cannot communicate with the web layer directly. The need for correct syntax formulation and subsequent submission to particular system layer components for processing is fundamental in the implementation of the mechanism in the web layer for processing the raw data entered by the user and submitting it to specific system components. The scope of the web layer covers transferring the user query and data to and from the system layer, verifying parameters and alerting accordingly, catching web-layer-specific errors and exceptions before the wrong query or data is sent to the system, file upload or download, file or script display functions, and so forth. Therefore the web layer is a publicly accessible abstraction for the remaining layers of WImpiBLAST. It isolates the system and resource details from users, hides the complexity of the individual resources and enables smooth interaction with the bioinformatics applications installed on the HPC cluster.

**1.1.2. Layer 2: The system and software layer.** The system layer is the functional core of WImpiBLAST, and is responsible for forwarding user queries to the requested resources after proper validation. This layer also houses the applications and configuration files necessary for processing user queries received from the web layer. The system layer enables additional validation of the user input, providing a logging facility and error handling that complements the features provided by the web layer. For instance, if the query submitted by the user through the web interface is syntactically correct but logically invalid, such as requesting more resources than available or using the wrong application program in a job script, then the error will not occur in the web layer; the system layer will either block such requests or terminate such job requests automatically, as soon as the resource manager encounters an invalid request for resources. Likewise, when the application detects that a user has requested an invalid program to be used against specific data types, the job will be terminated by the application environment and logged accordingly. In the context of WImpiBLAST, the scope of the system and software layer includes, but is not limited to, recording user requests, validating user queries and data, error capturing and logging, user validation and file handling. Therefore, the system layer acts as the core of the WImpiBLAST architecture.

**1.1.3. Layer 3: The HPC resource layer.** The resource layer is responsible for job execution. The resource layer and its components are accessible only through the cluster’s private network and can be accessed only from the master node of the HPC cluster. The benefit of this approach is that since all the legitimate users will have their account on the HPC cluster, the administrator can implement access policies, authentication policies and user-based privileges in the system layer to ensure fair use of resources. Also, this approach minimizes the user effort needed to understand the system, while administrators can configure access to system resources without the intervention of the user. Since the web layer is isolated from the resource layer, not every user who can access the web portal will have the same privileges as the priority users. After login to the web portal, all users will have a uniform view of the entire system. Jobs of high priority users will take precedence over other users depending on the back-end policies and rules implemented by the system administrator. However, users can always request to modify their resource usage limit or privileges. In the context of WImpiBLAST, the scope of the resource layer includes, but is not limited to, compute nodes, interconnects, storage nodes, high-speed Ethernet infrastructure, and so on. Hence the resource layer acts as the powerhouse of WImpiBLAST by actually executing computationally intensive queries.

### 2. Implementation

We have used Apache Struts 1.3 (http://www.tomcat.apache.org) for implementing the web interface. Apache Tomcat (http://www.tomcat.apache.org) is our default application server running on a 64-bit Centos 6.2 (http://www.centos.org) system. For resolving user permission issues in some modules, the ganymed application programming interface (build 250) for Java (https://code.google.com/p/ganymed-ssh-2/) was used and for client side validations the jqueryui (http://www.jqueryui.com) was used. WImpiBLAST requires the installation of the Java Development Kit (JDK) (http://www.oracle.com/technetwork/java) for the execution of the Java programs used for validation of inputs and email communications. We have used Java 1.7.0, but a higher version of the JDK should also work without any issues. Torque is the default resource manager for job submission on the HPC cluster and we have tested our implementation with Torque versions 3.0.2 and 3.0.5. We have tested our implementation only with Centos 6.2 and Apache Tomcat 7.0.42. The current implementation allows only registered users of the system (users having an account on the HPC system) to log in and perform tasks. Users can create and modify job scripts and submit jobs remotely through the web interface. Also, users can upload their data to the server, download result files or scripts, and view result files in the web browser to track the progress of running jobs. Additionally, users can also view or manage their jobs.

### 3. Procedural Workflow

**Figure 2:**
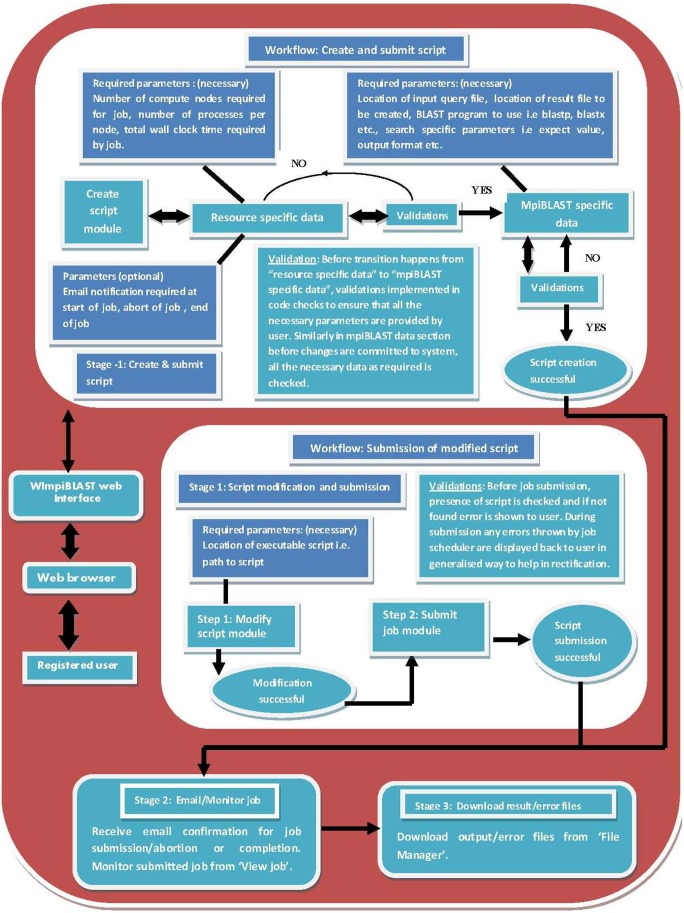
Procedural workflow in WImpiBLAST showing the initiation of mpiBLAST job, the input required at each stage and validations between stages to prevent invalid data from entering into successive stages.

This section discusses the workflow involved in using WImpiBLAST, describing the initiation of user requests from the web layer, the subsequent handling and processing at the system layer and the final allocation to the resource layer for execution. Users will need to follow a basic one-step process to initiate their first job through WImpiBLAST. A concise representation of the job initiation process is shown in Figure 2, which shows the inputs involved at different stages in job initiation and the respective validations implemented between stages. Once a given stage completes its processing of the input data, it subsequently pipes the output to the next stage to complete the process; this inter-stage data validation is also implemented in WImpiBLAST. If the output generated by a stage is not valid, or if some data is missing, then it can’t be piped into the next stage until the user resolves all the necessary data conflicts as indicated on the interface.

#### 3.1. Creation and submission of a new script

**Figure 3:**
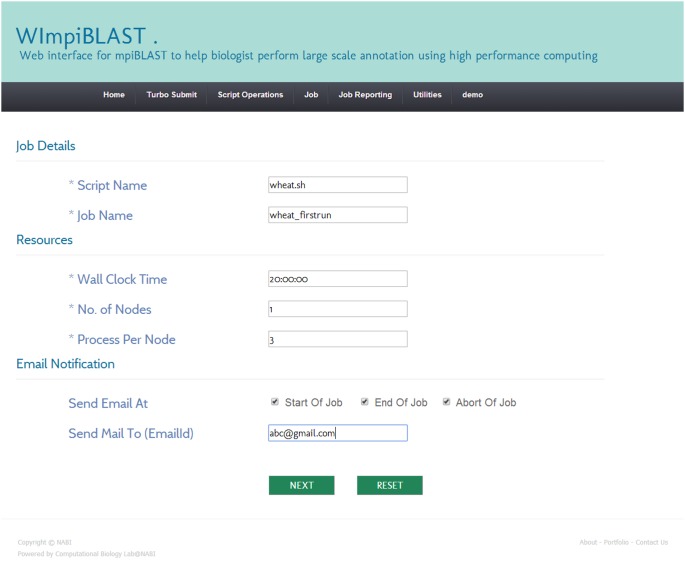
Snapshot of computational resource specific section of script creation module.

**Figure 4:**
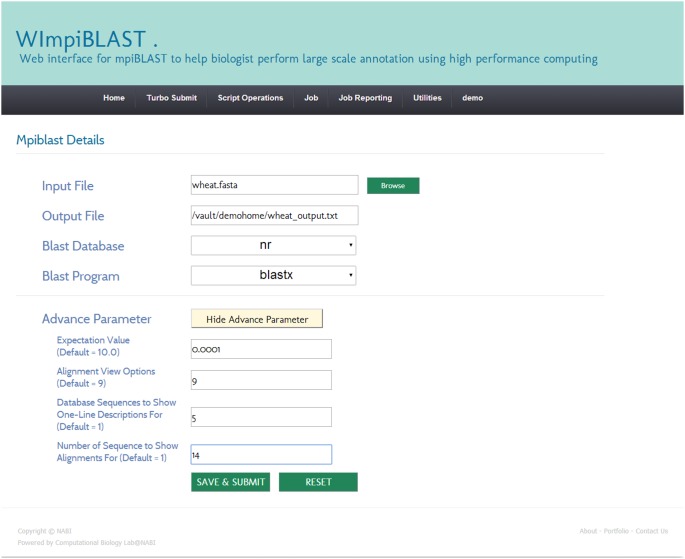
Snapshot of mpiBLAST specific section of create script module.

A script is an executable file that contains details related to the requested resources (hardware and software) and mpiBLAST-specific information. The resource-specific section may contain the number of required computing resources, the total amount of time required by the job, email-related information, and so forth. The mpiBLAST-specific section may contain information such as the location of the FASTA-format query file, the location of the result file, the BLAST program to use, and so on. Users can easily create a script through the script operations menu and then submit it for execution. A screenshot of the script creation module in WImpiBLAST is shown in Figures 3 and 4.

#### 3.2. Illustrative sample script

**Figure 5:**
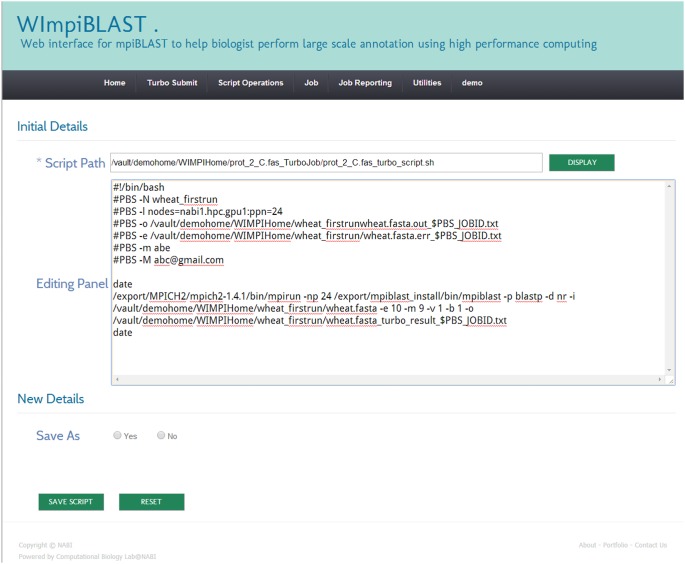
Sample created script.

A sample script created through the web interface is shown in Figure 5.

#### 3.3. Submission of modified script

**Figure 6:**
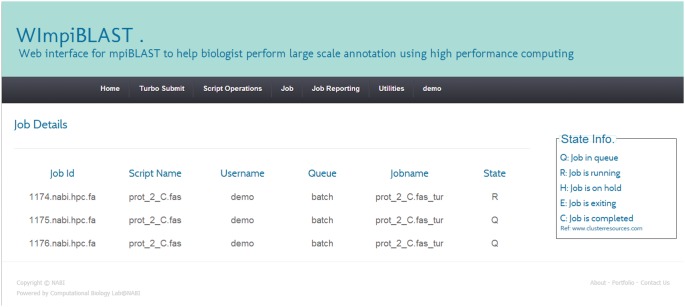
Snapshot of Job reporting module.

Once a script has been created, it can be customized for new jobs by making minor changes through “Modify Script” option and submitted through the “Submit Job” option. Submission of scripts to the HPC cluster is done through the Torque resource manager. We selected Torque because of its widespread usage, its open source advantage and due to the past experience of our institutional users with Torque. If the script submission is successful then the user can easily view or manage their jobs through the web interface. A screenshot of the job reporting module is shown in Figure 6.

#### 3.4. File manager utility

**Figure 7:**
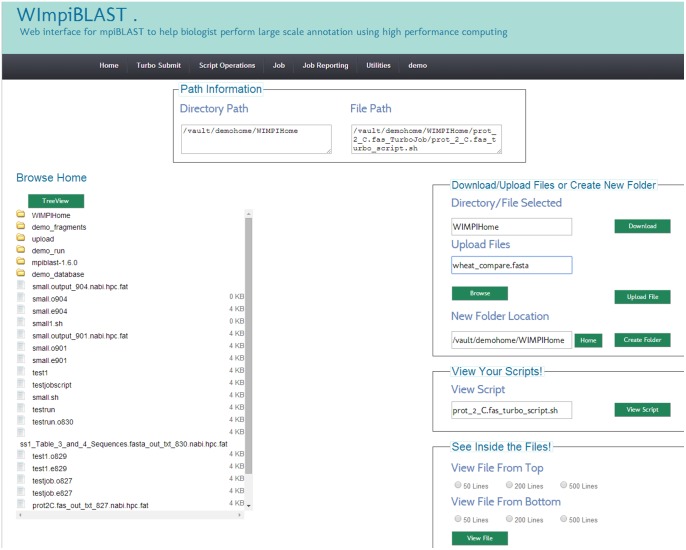
Snapshot of File Manager module showing user home directory in the file system and options of download/upload/view for files.

The unique TreeView feature shown in Figure 7 was implemented for browsing the userspace filesystem. The files and directories are automatically sorted by their last modification date, and the file size is displayed beside each file. The option of downloading files and directories is available to allow the user to download results and other files.

### 4. Installation

Installing WImpiBLAST requires the Apache Tomcat server that comes with almost all Linux distributions. However, the Torque resource manager should be installed on the system to allow the submission, monitoring and management of jobs from the web interface (http://www.adaptivecomputing.com). The administrator only needs to copy the WImpiBLAST.war file to the ‘webapps’ directory of the Apache Tomcat server and set the parameters in the configuration file to get started with the WImpiBLAST interface.

### 5. Administration and Security

This section describes the administration modules in WImpiBLAST and the https secure communication for login. The administration modules are currently undergoing beta testing and are not included with the existing WImpiBLAST package. The administration section of WImpiBLAST is isolated from the user section, and the administrator has unrestricted access to all jobs in the system. The administration modules are activated when the root user logs in from the web interface. The modules accessible to root cannot be accessed by a non-root user. Administrators can view, delete, hold, and release jobs from all users or a specific user by filtering the jobs by username. The administration section also provides a facility for viewing, in a web browser, a comprehensive job history for any current or previously submitted job.

User management is done by the system administrator, who can manually add and delete users and modify user settings such as their default permissions and privileges through command line. Users can use the same username and password for logging in to both the WImpiBLAST portal and the HPC system.

### 6. Manuals and Demonstration Server

Users can refer to the comprehensive user manual (Text S1) for in-depth descriptions of the functioning and handling of the different modules of WImpiBLAST. The WImpiBLAST package also includes an installation manual (Text S2) and a quick start guide (Text S3) to help initiate specific tasks. A demonstration server can be accessed at http://wimpiblast.nabi.res.in; it is specifically intended to help researchers or users understand the utility, robustness and simplicity of the interface. We encourage users to download the stable release of WImpiBLAST and deploy it at their respective HPC clusters to facilitate use by biologists.

## Results and Discussion

This section discusses use cases by executing NCBI BLAST+ and mpiBLAST searches on an in-house large nucleotide database and an non redundant (NR) protein database (downloaded from NCBI) through the command line, other web interfaces and WImpiBLAST. We have summarized the experimental findings, details of the search databases and the specifications of the systems used during the experiment in the form of tabular data.

In our use case experiment we used a symmetric multiprocessor (SMP) server having 48 cores and our in-house HPC cluster having 448 cores. Details of databases, programs and computing systems can be found in Table S1 and Table S2. We executed three use cases to observe the performance of the application under different test conditions such as size of search database, number of aligned sequences, number of cores and so on, as described in following paragraphs. Tests were performed under ideal system load when no other compute-intensive jobs were running on the SMP server or HPC cluster in order to assess the best performance gain achieved by the application. Also, multiple tests were executed and the readings shown in the tables are the best obtained during multiple runs of each use case instance. Appropriate data-intensive scientific computing questions are posed at the beginning of each use case for better understanding of the analysis acceleration.

### 1. Use Case 1

Q1. Can mpiBLAST be used for data mining in large nucleotide databases?

An in-house large database was developed by merging wheat RNA-seq data from 81 sequencing runs, downloaded from the NCBI Sequence Read Archive (SRA) database. The total size of this merged database was 272 GB. We wanted to perform data mining in this large nucleotide database for sequences that have similarity with the genes of interest. The requirement to filter these sequences was important for the subsequent analysis to identify non-synonymous variation and for allied analysis. We used 12 nucleotide sequences of varying length as query sequences to find the turnaround time in searching the large database. The large nucleotide database was used and the number of aligned sequences varied from 1 to 100000 by keeping other run-time parameters such as output format or expected value constant. NCBI BLAST+ was executed on an SMP server through the Sequenceserver graphical user interface (GUI) with the number of threads set to the total number of available cores, i.e. 48, and mpiBLAST was executed in the HPC cluster through WImpiBLAST with the number of cores set to 192. We used the maximum number of threads for NCBI BLAST+ to find the maximum core utilization and speed-up possible on the SMP server. However, for mpiBLAST we restricted our execution to only 192 cores because we observed saturation in the time gain of mpiBLAST if the number of cores increased beyond 192 in our HPC cluster. We concluded that if individual sequences were to be searched against a large database then increasing the number of cores beyond the threshold value of 192 cores does not provide any further gain in performance in terms of turnaround time on our HPC cluster. It was observed that mpiBLAST was 16 times faster than NCBI BLAST+ for aggregate average run times of all queries (Table 4). However, for large numbers of aligned sequences, e.g. 100000, we executed searches with the longest and shortest sequences only. It was observed that if the number of aligned sequences is large then mpiBLAST outperforms NCBI BLAST+ by roughly 51 times for sequence 6 (849 base pairs in length) and approximately 1000 times for sequence 2 (4857 base pairs in length). Since the data mining in question involved a huge database of around 1.4 billion sequences, we limited the number of aligned sequences to 100000 to make a fair estimation of the time required to achieve BLAST search results for such a computationally intensive task (Table 5).

**Table 4:** Turnaround time computed using Sequenceserver on SMP server and WImpiBLAST on HPC cluster.

*Query Sequences*	*Turnaround time using NCBI blastn* *through Sequenceserver;* *no. of threads = 48*	*Turnaround time using mpiBLAST* *through WImpiBLAST;* *no. of cores = 192*
Sequence 1(base pairs = 2576)	3.17 minutes	18 seconds
Sequence 2(base pairs = 4857)	5.55 minutes	23 seconds
Sequence 3(base pairs = 3749)	5.05 minutes	15 seconds
Sequence 4(base pairs = 4407)	3.37 minutes	21 seconds
Sequence 5(base pairs = 1643)	4.07 minutes	12 seconds
Sequence 6(base pairs = 849)	5.11 minutes	08 seconds
Sequence 7(base pairs = 2132)	4.03 minutes	10 seconds
Sequence 8(base pairs = 2121)	3.13 minutes	12 seconds
Sequence 9(base pairs = 2194)	3.16 minutes	12 seconds
Sequence 10(base pairs = 1683)	3.21 minutes	09 seconds
Sequence 11(base pairs = 1117)	5.18 minutes	12 seconds
Sequence 12(base pairs = 1336)	3.52 minutes	11 seconds

mpiBLAST achieved the highest speedup in case of sequence 6 where it outperforms NCBI BLAST+ by roughly 38 times but on average mpiBLAST performed 16 times faster than NCBI BLAST+ for aggregate run times of all queries. (*No. of aligned Sequences = 1; No. of sequences = 12; Type: Nucleotide; Average base pair length: 2389; Query sequences file: ss1_Table_4_and_5_Sequences.fasta* (Text S4)*; Search database: In-house large nucleotide database (size = 246 GB after formatting)*).

**Table 5:** Turnaround time computed using NCBI BLAST+ on SMP server and mpiBLAST on the HPC cluster.

*Query Sequences*	*Turnaround time using NCBI blastn;* *no. of threads = 48*	*Turnaround time using mpiBLAST;* *no. of cores = 192*
Sequence 2(base pairs = 4857)	5.26 hours	18 seconds
Sequence 6(base pairs = 849)	25 minutes	29 seconds

It was observed that if a number of aligned sequences is large then mpiBLAST outperforms NCBI BLAST+ by roughly 51 times for sequence 6 (849 bp in length) and by roughly 1000 times for sequence 2 (4857 base pairs in length). (*No. of aligned Sequences = 100000; No. of sequences = 2; Type: Nucleotide; Average base pair length: 2853; Query sequences file: ss1_Table_4_and_5_Sequences.fasta* (Text S4)*; Search database: In-house large nucleotide database (size = 246 GB after formatting)*).

### 2. Use Case 2

Q2. How much acceleration can be achieved for large-scale transcriptome annotation by mpiBLAST on an HPC cluster?

To annotate a large FASTA-format file (having 43758 transcriptome contigs) we used mpiBLAST and the NCBI BLAST+ program at a different number of cores to search the NR protein database. The NCBI BLAST+ program was executed on an SMP server through the command line with the number of threads set to the total number of available cores, i.e. 48, and mpiBLAST was executed in the HPC cluster through WImpiBLAST by using 48 and 448 cores respectively. It should be noted that unlike Use Case 1, where we observed saturation beyond 192 cores for mpiBLAST, in this case turnaround time was decreasing considerably with increase in the number of cores, so we employed all 448 cores to find maximum time reduction. In this case mpiBLAST was 1.76 times faster than NCBI BLAST+ when both used the same number of cores, i.e. 48. However, on scaling mpiBLAST to full capacity, i.e. 448 cores, it outperformed NCBI BLAST+ by roughly 23.03 times. The findings of Use Case 2 are summarized in Table 6.

**Table 6:** Turnaround time computed using NCBI BLAST+ on SMP server and mpiBLAST on the HPC cluster.

*Program Used*	*NCBI blastx command* *line execution;* *no. of threads = 48*	*mpiBLAST through* *WImpiBLAST;* *no. of cores = 48*	*mpiBLAST through* *WImpiBLAST;* *no. of cores = 448*
Turnaround time	96.96 hours	55 hours	4.21 hours

The readings show that on the same number of cores i.e. 48, mpiBLAST outperforms NCBI BLAST+ by roughly 1.76 times. The maximum speedup was observed when using all 448 cores on the HPC cluster where mpiBLAST is 23.03 times faster than NCBI BLAST+. (*Number of sequences in fasta file = 43758; Type: Nucleotide; No. of aligned Sequences = 1; NCBI BLAST+ executed on SMP server and mpiBLAST executed on HPC cluster; Query sequences file: ss2_Table_6_Sequences.fasta* (Text S4)*; Search Database: NR protein database (downloaded from NCBI)*).

### 3. Use Case 3

Q3. Can mpiBLAST accelerate transcriptome annotation on a single SMP server where the number of cores is constant?

We decided to directly compare the performance of NCBI BLAST+ and mpiBLAST by running both applications on the SMP server. This test was performed to eliminate any scope for biased readings due to the superiority of the underlying hardware architecture. Since the system hardware such as processor, memory and so on are kept constant and the run-time parameters are constant too, this case strongly corroborates the findings described in Use Case 1 and Use Case 2 showing better performance by mpiBLAST. We kept the number of aligned sequences the same, i.e. 1, and used a randomly created input file (having 1000 contigs) extracted from the FASTA file used in Use Case 2. In this case also, mpiBLAST was 1.2 times faster than NCBI BLAST+ program. The findings are summarized in Table 7. Although the gain is moderate, this result makes an excellent case for biologists who want to search databases of a similar size to NR for large transcriptome query files using a limited number of cores achieving a time reduction by using mpiBLAST.

**Table 7:** Turnaround time computed using NCBI BLAST+ and mpiBLAST on SMP server.

*Program used*	*NCBI blastx command line* *execution; no. of threads = 48*	*mpiBLAST through WImpiBLAST;* *no. of cores = 48*
Turnaround time	4.17 hours	3.55 hours

The readings show that on the same SMP server and a similar number of cores i.e. 48, mpiBLAST outperform NCBI BLAST+ by roughly 1.2 times. (*Number of sequences in fasta file = 1000; Type: Nucleotide; No. of aligned Sequences = 1; Both NCBI BLAST+ and mpiBLAST executed on SMP server; Query sequences file: ss3_Table_7_Sequences.fasta* (Text S4); *Search Database: NR protein database (downloaded from NCBI)*).

It should be noted that in Use Case 2 and Use Case 3, BLAST+ searches were executed through the command line and not via the Sequenceserver GUI due to a limitation of the text area in Sequenceserver. Also, in Use Case 1 the reading obtained for Sequenceserver is not the actual processing time, but the total time to execute BLAST searches *and* perform input/output, i.e. taking sequences from the text area, processing them on the system and serving the results back on the web page. However, this does not affect the accuracy of the readings drastically because the time for input/output was negligible when compared to the actual processing time.

We have not come across any open-source portal or web interface which enables biologists to submit parallel BLAST sequence searches to an HPC cluster. G-BLAST [17], a grid-based solution for mpiBLAST on computational grids, has not been widely used due to the unavailability of the source code in the public domain. In WImpiBLAST, we learned from our predecessors and developed a solution that can be set up for small to medium-sized HPC clusters with the least effort by administrators as well as being easy to use by biologists who need to expedite the annotation part of their research.

In this rapidly advancing era of genomics, due to the tremendous computational demand for processing the data, algorithms ported to General-Purpose computing on Graphics Processing Units (GPGPU) [18] and Field-Programmable Gate Arrays (FPGA) [19] hardware are being actively explored to accelerate analysis. New algorithms, such as USEARCH and UBLAST [20], have become very popular and are being used by thousands of users worldwide. CloudBLAST [21], a Hadoop-based implementation using the MapReduce paradigm, is suitable for analyzing the massive volume of NGS data by effectively storing and processing large data files in a scalable, cost-effective and resilient manner. We strongly believe that such innovative annotation acceleration solutions would be used more by biologists when delivered through a simple-to-use interface like WImpiBLAST.

## Future Work and Conclusion

Future work related to WImpiBLAST can progress in several directions related to interface richness. The current implementation focuses primarily on the mpiBLAST application and uses distinct but well-connected modules to execute parallel applications on an HPC cluster. In future, more parallel bioinformatics applications will be integrated in WImpiBLAST framework. Currently only users registered on the master node of the cluster can submit jobs through WImpiBLAST, but we are trying to integrate user mapping mechanisms such as Light Weight Directory Access Protocol (LDAP) to allow users who do not have an account on the HPC cluster to submit parallel jobs; this will increase the penetration rate of WImpiBLAST among researchers. The administration modules are currently limited to job monitoring and management or tracing job history, but in future the administrator will be able to create queues, adjust resource reservation or adjust user priority through the web interface itself. The current installation process is not entirely platform independent due to resource manager and application dependencies. We will consider integrating support for the Distributed Resource Management Application API (DRMAA) and EasyBuild or a similar tool in WImpiBLAST for making the job management and application installation process more flexible.

This paper presents the open-source WImpiBLAST web interface for mpiBLAST and discusses in detail the design decisions taken during its development, its robust architecture and use cases. The architecture adopted in this project can also be used as a template for porting any scientific computing application. The user-friendly WImpiBLAST interface will facilitate and encourage the use of supercomputing resources by biologists. This research will have a direct impact on the rate of knowledge discovery by accelerating various large-scale annotation projects.

### Availability and Requirements

Project name: WImpiBLAST

Demonstration Server: http://wimpiblast.nabi.res.in

Download Link: http://code.google.com/p/wimpiblast/

Platform: Linux

Programming Language: Java

Framework: Struts-1.3.0

Other Requirements: Apache-tomcat-7.x.x (www.tomcat.apache.org) and Torque resource manager (www.adaptivecomputing.com).

## Supporting Information

Table S1 Database and program related information as used during the use case tests.
(DOCX)

Table S2 System specific information used in Experiment.
(DOCX)

Text S1 User Manual.
(PDF)

Text S2 Installation Manual.
(PDF)

Text S3 Quick Start Guide.
(PDF)

Text S4 Hyperlinks to fasta file containing sequences used in use case runs.
(DOCX)
