# Supplementary material for: WImpiBLAST: Web Interface for mpiBLAST to Help Biologists Perform Large-Scale Annotation Using High Performance Computing
Source: PLoS One. 2014 Jun 30;9(6):e101144. doi: 10.1371/journal.pone.0101144 (PMC4076281; doi:10.1371/journal.pone.0101144)
Supplement: Table S1 — Database and program related information as used during the use case tests. (DOCX) [file pone.0101144.s001.docx]

**Supplementary Table 1:** Database and program related information.

| **Relevant Database and Program Information** | | |
| --- | --- | --- |
| **Search database** | In-house large database | Non-Redundant (NR) protein database |
| **Database Type** | Nucleotide | Protein |
| **Source** | In-House (developed by merging wheat RNA-Seq data of 81 sequencing runs, downloaded from SRA database of NCBI) | NCBI website (ftp://ftp.ncbi.nlm.nih.gov/blast/db/FASTA/nr.gz) |
| **Fasta File size (Unformatted)** | 272 GB | 18 GB |
| **Formatted Database Size** | 246 GB | 24 GB |
| **Number of Database Fragments** | 100 | 12 |
| **Program** | Blastn | Blastx |
| **No. of sequences** | 1476605950 | 31601460 |
| **mpiBLAST version**: 1.6.0 | | |
| **NCBI blast+ version**: 2.2.28+ | | |
| **WImpiBLAST version**: 1.0.0 | | |
| **SequenceServer version**: 0.8.6 | | |
