# Supplementary material for: WImpiBLAST: Web Interface for mpiBLAST to Help Biologists Perform Large-Scale Annotation Using High Performance Computing
Source: PLoS One. 2014 Jun 30;9(6):e101144. doi: 10.1371/journal.pone.0101144 (PMC4076281; doi:10.1371/journal.pone.0101144)
Supplement: Table S2 — System specific information used in Experiment. (DOCX) [file pone.0101144.s002.docx]

**Supplementary Table 2:** System specific information of use cases.

| **System Configurations** | | |
| --- | --- | --- |
| **Systems used** | SMP node | HPC cluster |
| **No. of cores available** | 48 | 448 |
| **Processor** | AMD Opteron Processor 6176 | Intel(R) Xeon(R) CPU E5-2670 |
| **Clock speed** | 2.30 GHz | 2.60 GHz |
| **RAM** | 252 GB | 63.02 GB per node |
| **OS** | Open Source Centos-6.4 | Open Source Centos-6.2 |
