# Supplementary material for: WImpiBLAST: Web Interface for mpiBLAST to Help Biologists Perform Large-Scale Annotation Using High Performance Computing
Source: PLoS One. 2014 Jun 30;9(6):e101144. doi: 10.1371/journal.pone.0101144 (PMC4076281; doi:10.1371/journal.pone.0101144)
Supplement: Text S1 — User Manual. (PDF) [file pone.0101144.s003.pdf]

NABI

National Agri-Food Biotechnology Institute

WImpiBLAST Portal

User Manual

Table of Contents

Chapter 1

Introduction

1.1 Preface .... [6]

1.2 Salient features and benefits .... [7]

1.3 Advantages .... [8]

1.4 Flowchart description of WImpiBLAST portal .... [9]

Chapter 2

WImpiBLAST Portal Overview

2.1 Login page .... [10-11]

Chapter 3

Portal User's Area

3.1 Script operations

3.1.1 Script operations .... [12]

3.1.1.1 Create and Submit New Script .... [13-18]

3.1.1.2 Modify Older Script ………………………………………………………………………………………… [19]

3.2 Job

3.2.1 Submit Scripts ……………………………………………………………………………………………… [21-26]

3.3 Job Reporting ……………………………………………………………………………………………………… [27]

3.3.1 Job Monitoring and Management ……………………………………………………………………….. [27]

3.4 Job Management ………………………………………………………………………………………………….. [30]

Delete …….. [30]

Hold ……….. [30]

Release……. [30]

3.5 Utility …… [32]

3.5.1 Upload/Download Functionality …………………………………………………………………….. [34]

3.5.2 Tree View Functionality ………………………………………………………………………………….. [35]

3.5.3 View Script Functionality…………………………………………………………………………………. [36]

3.5.4 See Inside Files Functionality…………………………………………………………………………… [37]

3.5.5 Path Information Functionality………………………………………………………………………. [38]

3.6 Manual…….. [39]

WImpiBLAST Portal User Manual…………………………………………………………………………………… [39]

3.7 Miscellaneous features…………………………………………………………………………………………... [40]

3.7.1 Input Validation Functionality…………………………………………………………………………... [41]

3.7.2 File Size Validation Functionality…………………………………………………………………….. [42]

3.7.3 User alert Functionality……………………………………………………………………………………. [43]

3.8 Logout …… [44]

Appendix………. [45]

End of Manual……………………………………………………………………………………………………….. [46]

New Features

Following new features have been added in WImpiBLAST.

1. **New Turbo job feature**: Turbo Submit reduces the script creation and submit time cycle manifold, now users only need to browse for their input query file and Click on 'Turbo Submit'. Explore 'Turbo Submit (beta)'.

2. **Experience the Organized Job Directory Structure:** Now, with each successful job, a dedicated directory is created for holding all the files for that job. The directory bears the same name as jobname given by user, to help in remembering the particular files. The jobs directories are created inside 'WIMPIHome' directory of user's home.

3. **Find Script name Information of Jobs:** The script associated with each job is now displayed in "View Job". Helps you to remember which scripts are associated with the running jobs.

4. **Explore New Way of Browsing Files/Folders:** All the files are now sorted by last modification time, so you don't have to look for newly created files every time you submit a job. You will find new files/folders at the top of Tree View. Additionally size of each file is now displayed besides the file to help in assessing the progress of result. Explore utilities --> File Manager --> Tree View.

5. **Create & Download Folder:** Now you can create your own folder to house different files or data at one place. Also, just click on any folder to download it in compressed form. Explore Utilities --> File manager --> Tree View --> Create Folder.

6. **Find Workflow Diagram on Every Page:** A workflow diagram to let you understand how to submit scripts.

Introduction

1

1.1 Preface

In HPC Clusters, low level software's such as 'Job Schedulers' are used to ensure proper usage of diverse hardware and software resources, Job schedulers provide low level commands to submit computationally demanding tasks to the 'Resource Manager' for transparent distribution of the associated workload. The user can submit batch jobs, interactive jobs, and parallel jobs to the resource manager through job schedulers.

WImpiBLAST Portal is a user-friendly job submission, management and monitoring interface that is compatible with open source Job scheduler and resource manager i.e. Torque. WImpiBLAST is a web UI developed specifically for mpiBLAST. It is developed to give a user-friendly interface for molecular biologist to submit mpiBLAST job on HPC cluster.

Routinely most of the molecular biologist use NCBI portal for doing sequence similarity search for their gene of interest. With the advent of Next Generation Sequencing Technologies it has now become possible to study gene expression at Genome wide scale through RNA-seq experiments. Annotation of all the genes in a given transcriptome is computationally very intensive job which can be accelerated by using High Performance Computing cluster.

To do this transcriptome annotation themselves, Molecular Biologist are often limited due to steep learning curve to gain skills of programming and command line usage. By developing WImpiBLAST we are trying to help molecular biologist to overcome this limitation by helping them use high performance computing cluster for computationally intensive annotation jobs through a simple web interface.

1.2 Salient Features and Benefits

**1. Web based remote access.**	**WImpiBLAST can be accessed from any location through web browser. No additional software is needed.**
**2. Personalized job list and job status information.**	**End users can easily manage and view their jobs.**
**3. Manage resources**	**Administrator decides what all resources are accessible by the user by making changes at system level policies.**
**4. Job management**	**User can manage their jobs individually. However, administrator can also track and manage if need be.**
**5. Email notification**	**Users can chose to receive notification when jobs begin, finish or fail, allowing them to be informed when any of the above events happens.**
**6. Customizable interface**	**WImpiBLAST can be customized as per the new Bioinformatics application requirement to support more mpi enabled applications.**

1.3 Advantages

- The current version of WImpiBLAST can scale to environments with hundreds of users. The scaling limit of WImpiBLAST is only limited by number of registered users on HPC cluster. The scaling capabilities are also dependent on hardware capacity and user management policies at system level. Typically, WImpiBLAST is closely tied with authenticated users and will allow only those users who have their account on HPC system to login.
- Built using JSP/Struts-1.3.0 an industry specification making the portal installation portable and scalable. Currently WImpiBLAST does not use any database or flat files thus making the installation even simpler.
- Integrating new Parallel Application and customizing the WImpiBLAST Portal is easy as only separate form for handling new application's input/output is required.
- WImpiBLAST hides the complexity of diverse Cluster resources, interfacing with hardware/software, resource manager interfacing from end users thus letting users/domain scientists to concentrate on their respective science without having to worry about several computational aspects.
- The WImpiBLAST Portal facilitates creation, submission and monitoring of the job though web browser. Hence the end users are free from the strenuous tasks of command line script creation, job submission and monitoring.
- As WImpiBLAST is closely coupled with HPC Cluster thus the Super user privileges are still applicable if 'administrator' logs into the portal. Administrator can monitor all the users, jobs submitted by the users and job history.
- Impact on existing network and cluster infrastructure is minimal: the WImpiBLAST Portal makes use of native JAVA features to carry out various tasks in isolation. Also, due to system level commands used in performing several functions the overall impact on server hardware is very optimal. WImpiBLAST portal thus reduces load on hardware by using a mix of native java feature and Linux commands.

1.4 Flowchart Description of WImpiBLAST Portal

User Configuration Flowchart

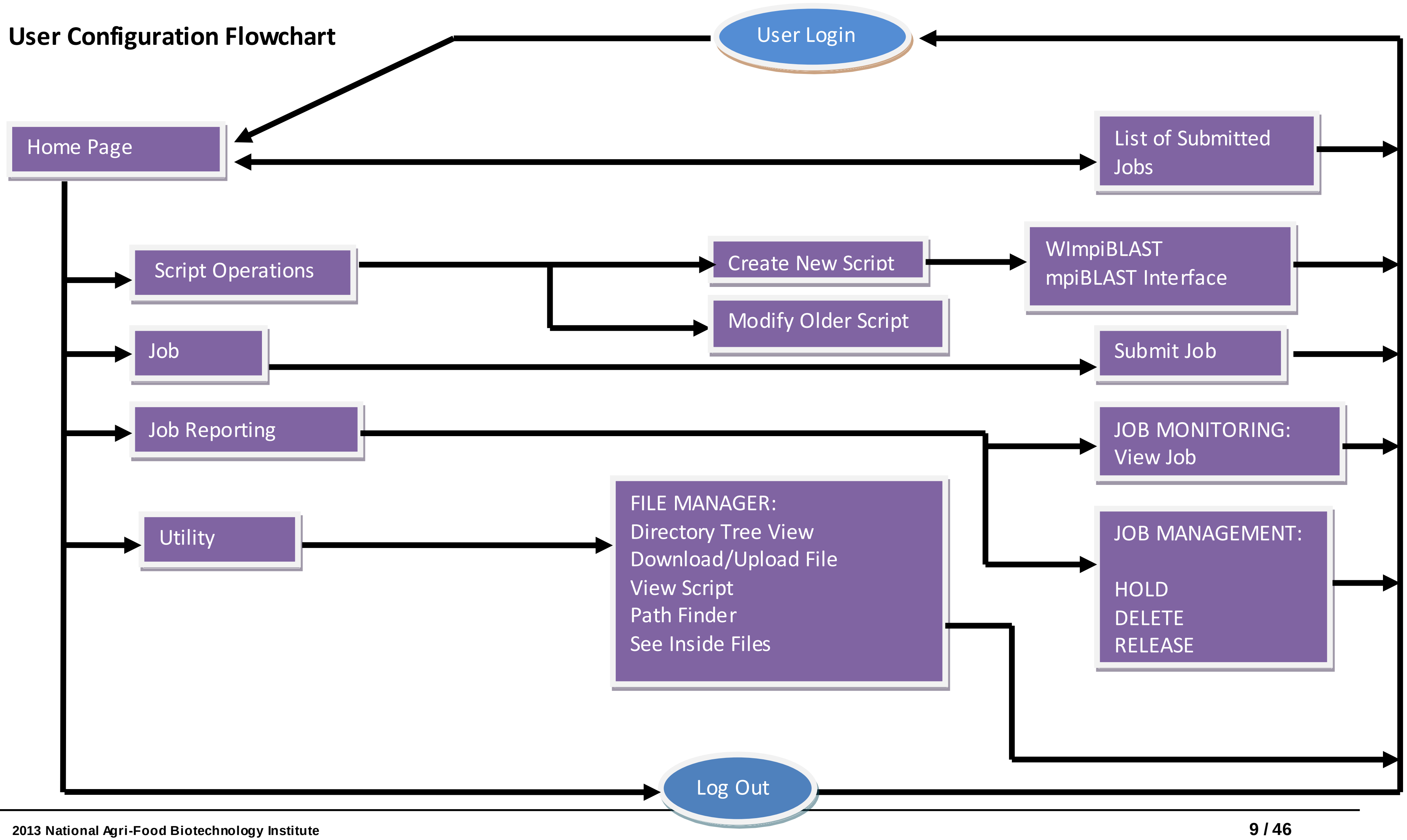

WImpiBLAST Portal Overview

2

2.1 Login Page

This Login page consists of login section and generic information about the features of WImpiBLAST portal.

From login page the user can also navigate to basic information section of WImpiBLAST portal such as “About” and “Portfolio” section using links given in the footer of the page.

2.1 Login Page Snapshot:

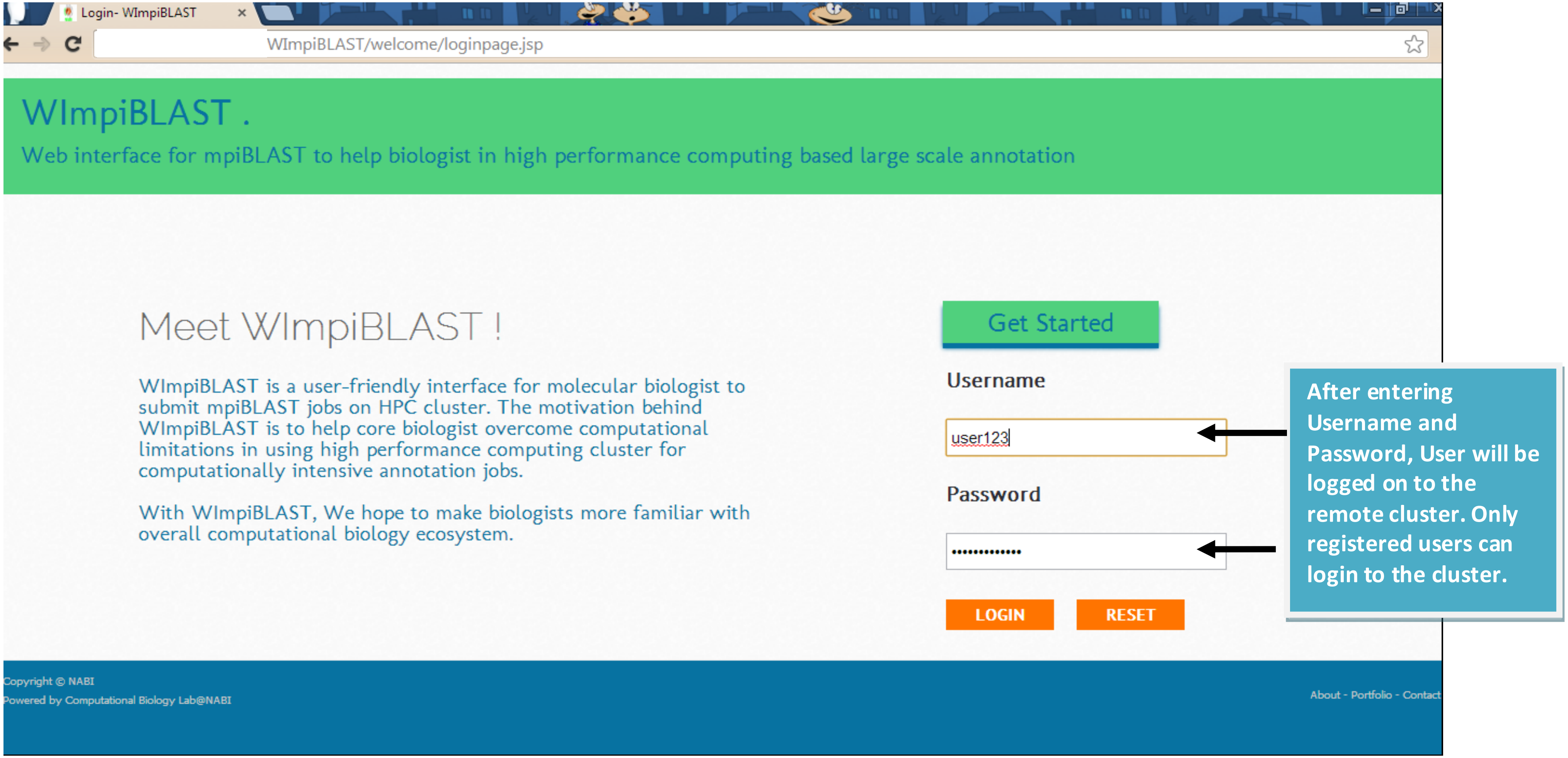

Figure: Login Page

User Area

3

3.1 Script operations

User is tasked with each of the following: The Job related functionality of WImpiBLAST portal includes following features:

- **Script Operations**
  - Create Script
  - Modify Script
- **Job**
  - Submit Script
- **Job Reporting**
  - View Job

Script Operations: This feature is sub divided further into create script and modify script modules. Create script provides a intuitive user interface by which user can create the job script online by providing all the relevant parameters. In modify script, user can modify or change previously created scripts and can also save the changed script by new name in the same location.

Job: This feature of WImpiBLAST portal includes a submit script user interface by which the end user can submit the job to the scheduler for execution. The submit script user interface first gathers the necessary information from the user such as number of nodes and wall clock time and then submit the job script for execution.

Job Reporting: This feature of WImpiBLAST portal is sub divided into view job and manage job modules. In short, Job reporting deals with providing the job specific details to the end user in an easy to read and understand format. View job will display only those jobs to the end user which are submitted by him/her in other words user 'A' can't view the jobs submitted by user B and vice versa. The view job module provides specific details for any particular job selected by user to view.

3.1.1 Create and Submit New Script

To run a batch job through Scheduler, you have to create a scheduler specific script using the corresponding prefix and prerequisites for the particular scheduler. The script specifies, among other things, the number of processors, the amount of memory, and the length of time you're requesting. Each system has additional resources that you can request.

Scheduler specific directives are specified as comments in the script. In particular, all lines beginning with say #PBS, in case if you are creating a PBS script, it is followed by the PBS directives. After the PBS directives is the body of the script -- commands which are executed when the script runs. In this way the script is created which is further submitted to the scheduler for scheduling based on system policies.

For E.g.:

<u>This is a sample PBS script</u>

For parallel jobs

Request 4 CPUS

#PBS -l ncpus=4

Request 1GB Memory

#PBS -l mem=1gb

```
# Request 3 hour 20 minutes of wallclock time (not CPU time!)

#PBS -l walltime=3:20:00

# Request that regular output and terminal output go to the same file

#PBS -j oe

# The following is the body of the script. By default PBS scripts execute in your home directory, not the directory from which they were submitted. The following line places you in the directory from which the job was submitted.

cd $PBS_O_WORKDIR

# Now we want to run the job "foo", which is an MPI job and

# is in the directory $PBS_O_WORKDIR. Note: mpirun defaults

# to 4 process, so we need to specify -np 4 to run on 4

# processors.

mpirun -np 4 ./foo
```

Create New Script Snapshot (fom-1)

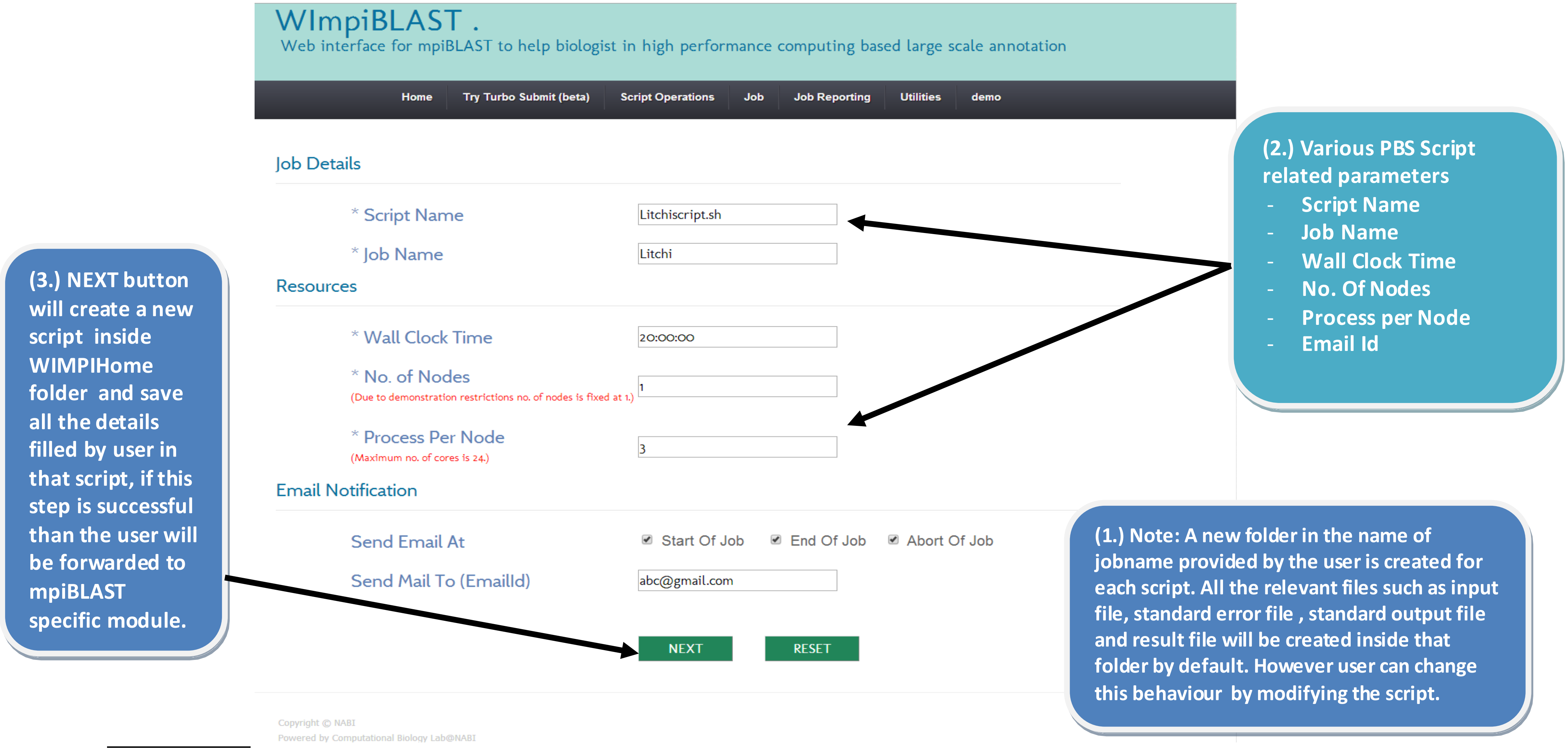

Figure: Create New Script

Create New Script Page Information or Parameters: The information present on create new script page is divided into four sub sections which are as follows:

Job reporting:
Script name
Job name

Resources
Number of nodes
Process per node
Wall clock time

Email/Notify:
Event to send email
Recipient email id

A brief description of above listed parameters is as follows:

Script name: This parameter requires the name by which the file will be created in the system after user clicks on NEXT button at the bottom of the page. Only alphabets (A-Z,a-z) are allowed in this field.

Job name: These parameters specify the name by which the Job will be submitted to the scheduler. This parameter is different from the filename parameter. For e.g. a job script can be present in the system by any user desired name such as “abc.sh” however if Job name field is “job” then the abc.sh script will be submitted by the name “job” to the scheduler. Only alphabets (A-Z,a-z) are allowed in this field. Every time user click on ‘NEXT’ button a new folder with jobname is created inside ‘WIMPIHome’ directory of user home. This folder will contain all the relevant files of the job such as result file, standard output file, standard error file and input query file etc.

Resources: This section of create script page consist of information related to typical hardware level resources which will be used for a script when it will be submitted to scheduler for execution. List of various parameters present in this sub section are as follows,

Number of nodes: This parameter specifies the total number of compute nodes that will be used for executing the script.

Process per node: This parameter specifies the total number of processes which will be simultaneously executed per node for execution of script.

Wall clock time: This parameter specifies the total amount of time allocated to the script for execution. For e.g. if the script execution crosses this allotted time interval than job scheduler will automatically suspend script execution and an email will be sent to the recipient email address.

Email/Notify: This field indicates the event on which you want to receive the notification email.

Create script application Section: This section contains blast specific parameters to be filled before the final script can be created for execution. Where Create Script Module creates script with all the job scheduler specific parameters the mpiBLAST section appends blast specific details into created script. This section can only be accessed after successfully completing the details of "Create Script module" otherwise user can not access this section of portal. The reason to link this section with creates script module is to maintain file consistency in the system and prevent users from unintentionally creating empty job execution scripts.

Description: You have to browse the input query file from the system by clicking on 'Browse' button. Once the file is selected, the input and output file paths are automatically updated by portal. Next you need to select blast database to search and blast program to use from dropdown menu. If you want to customize the search then fill the more parameters by clicking on 'Fill Advance Parameters' button to display the list of currently supported parameters.

3.1 mpiBLAST Application Section Snapshot

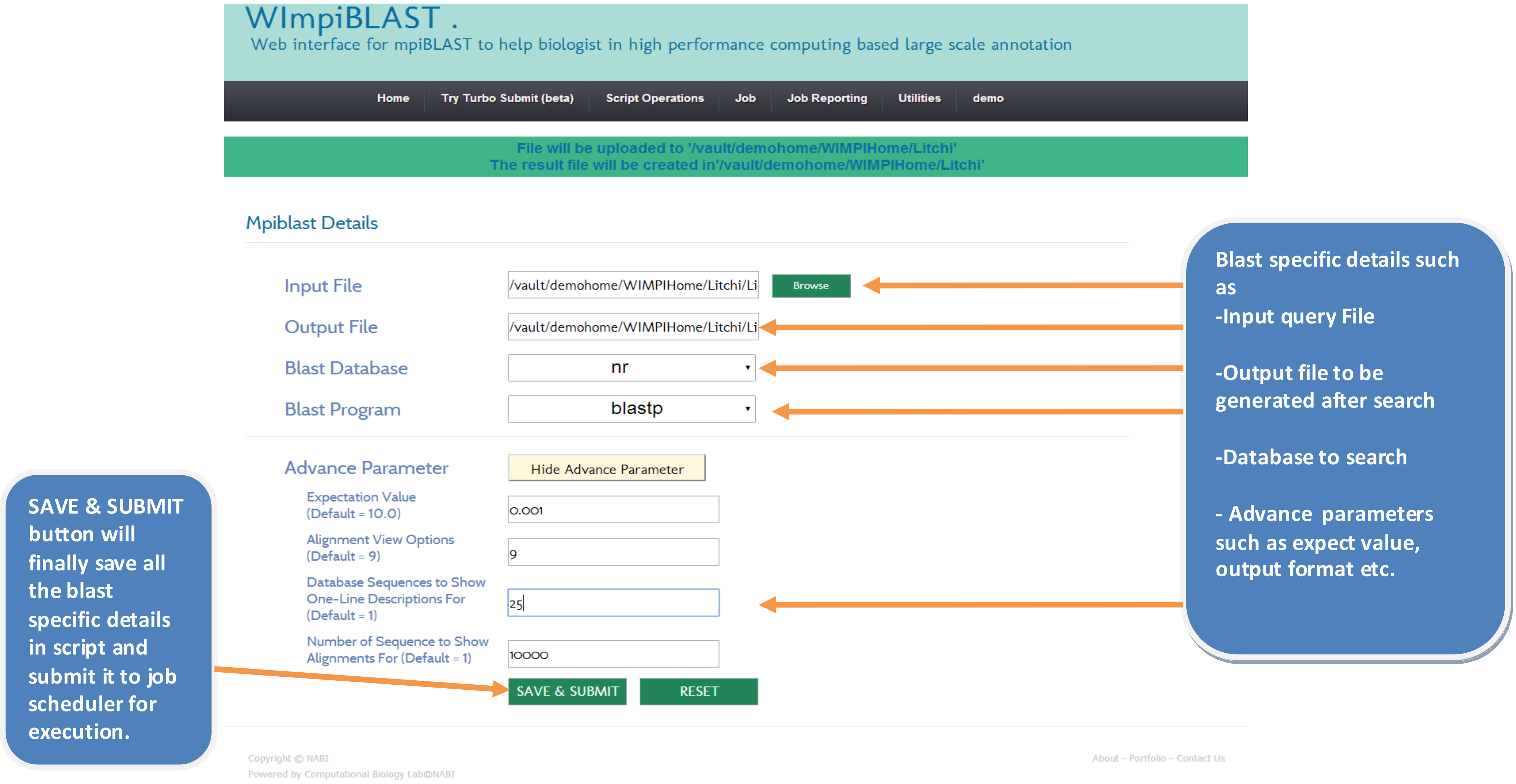

Figure: mpiBLAST application module

3.1.1.2 Modify older script:

The Modify script module provides the flexibility of changing the previously created scripts at any time and saving the modified script either by new name or by the same name thus overriding the older script. Modify script lets users change any specific parameters of the script or change the script entirely depending on the type of job requirement. Typically, there is no limit imposed by modify script on the degree to which the user can change the script as per the requirements.

Modify older script page information or parameters:

The information present on Modify Older script page is divided into four sub sections which are as follows

- Script path
- Editing panel
- Save as
- Save script as

A brief description of various parameters present on the modify script page is as follows:

Script path: This parameter specifies the path of the script that has to be changed or modified by the end user. The user can easily select the specified script by using the path finder feature of portal. When user successfully provides the path to the script, it is displayed in the text area with contents modifiable.

Editing panel: This area is reserved for displaying the script to the end user. Upon selection of the script for modification, the contents of that script are displayed in the area below script path label. User can easily change any parameter by directly making changes to the contents of the script or more parameters can be added by simply writing new command or directives among the already written contents.

Save as: This parameter specifies whether or not user wants to save the script as new file or not. User can simply choose to save the script as New File by selecting “Yes” or decline by selecting “No”.

Save script as: This parameter specifies the new name for the script. If this field is empty then the script will be saved by the same name i.e. its original name. In other words if new name and new location is not provided by the user then the original script will be over ridden.

Modify older script snapshot

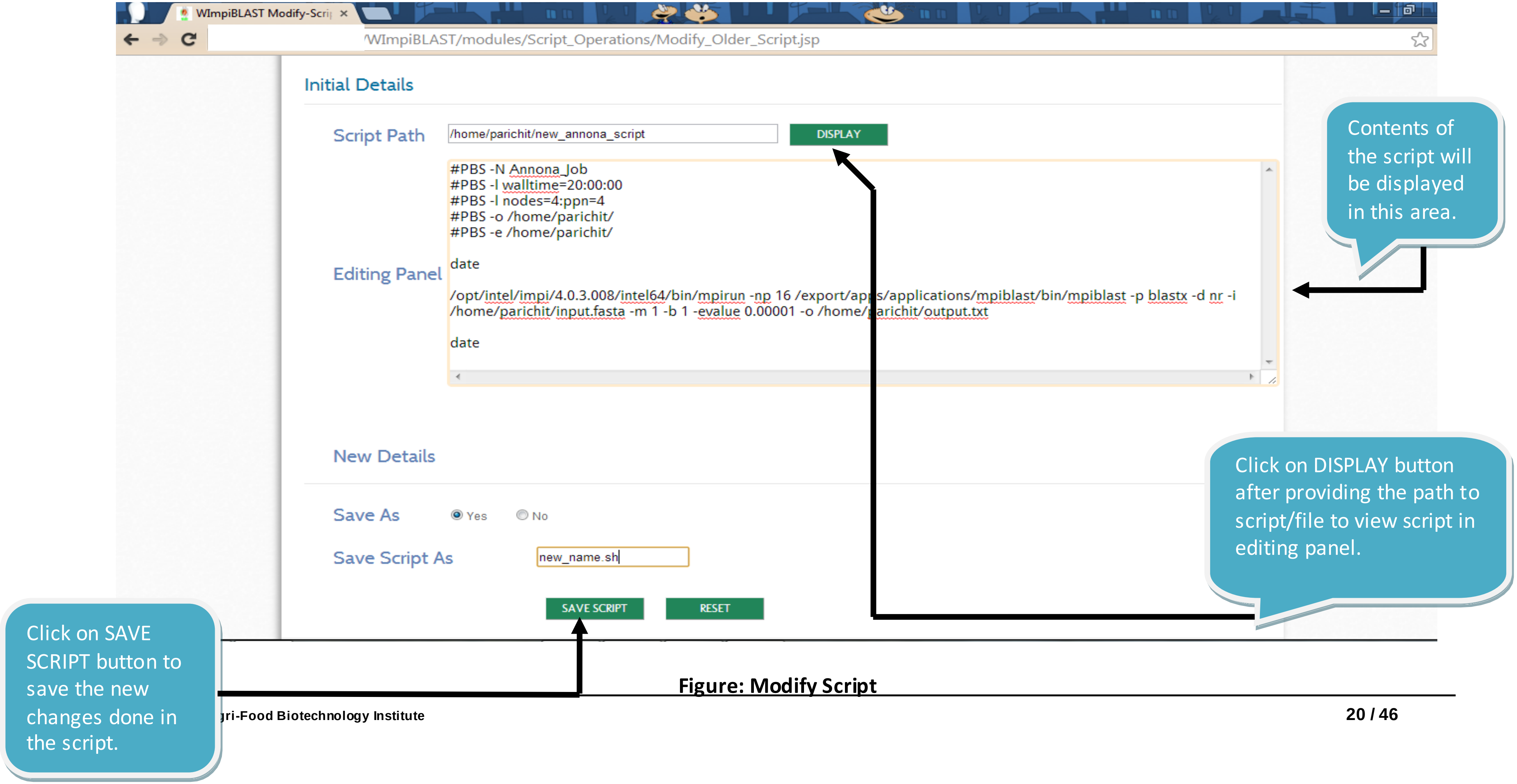

Figure: Modify Script

3.2 Job

Once user has set up scheduler environment, there is need to create a shell script file that contains the commands to run job. After creating a scheduler specific script using the corresponding prefix and prerequisites, user will have to submit this script to the scheduler by using the scheduler specific commands and flags. This process of submitting a job to the scheduler using the scheduler specific commands is referred as Job Submission.

With WImpiBLAST, since all the system level policies and setup of schedulers/configurations is done by system administrator hence user has no need to worry about setting up low level details to configure schedules or resource managers. Submit Job module of WImpiBLAST easily submits the job for user while also alerting for any error as encountered by job scheduler in starting the job.

The Submit Job module is subdivided on the basis of whether the user is submitting the script random script or not.

Random script: yes (For experts only)

Using the radio buttons present at the top section of module the user can select that whether the script that will be submitted is a random script or not. User can submit the script as random script in case where user wants to change various resource specific details with each execution of script for e.g. executing script by changing the number of nodes every time so in that case there is no need to change the script each time the number of nodes changes. By selecting the script as random script, the contents of the script will remain same but still it will execute with new parameters as submitted by user. This is also true for various other parameters as available in the random script menu of Job submission module.

Random script: No (Recommended)

However, it is expected that most of the time the user will be selecting “No” as selection for random script so that the only required parameter needed from user is path to the script.

Note: If the script is created using create script module of the portal then it is always advisable to select random script as “No”. In other words if you cannot decide what you should do with random scripts option than choose random script as “NO” and simply provide the path to already created script.

Submit job snapshot: Random script: Yes

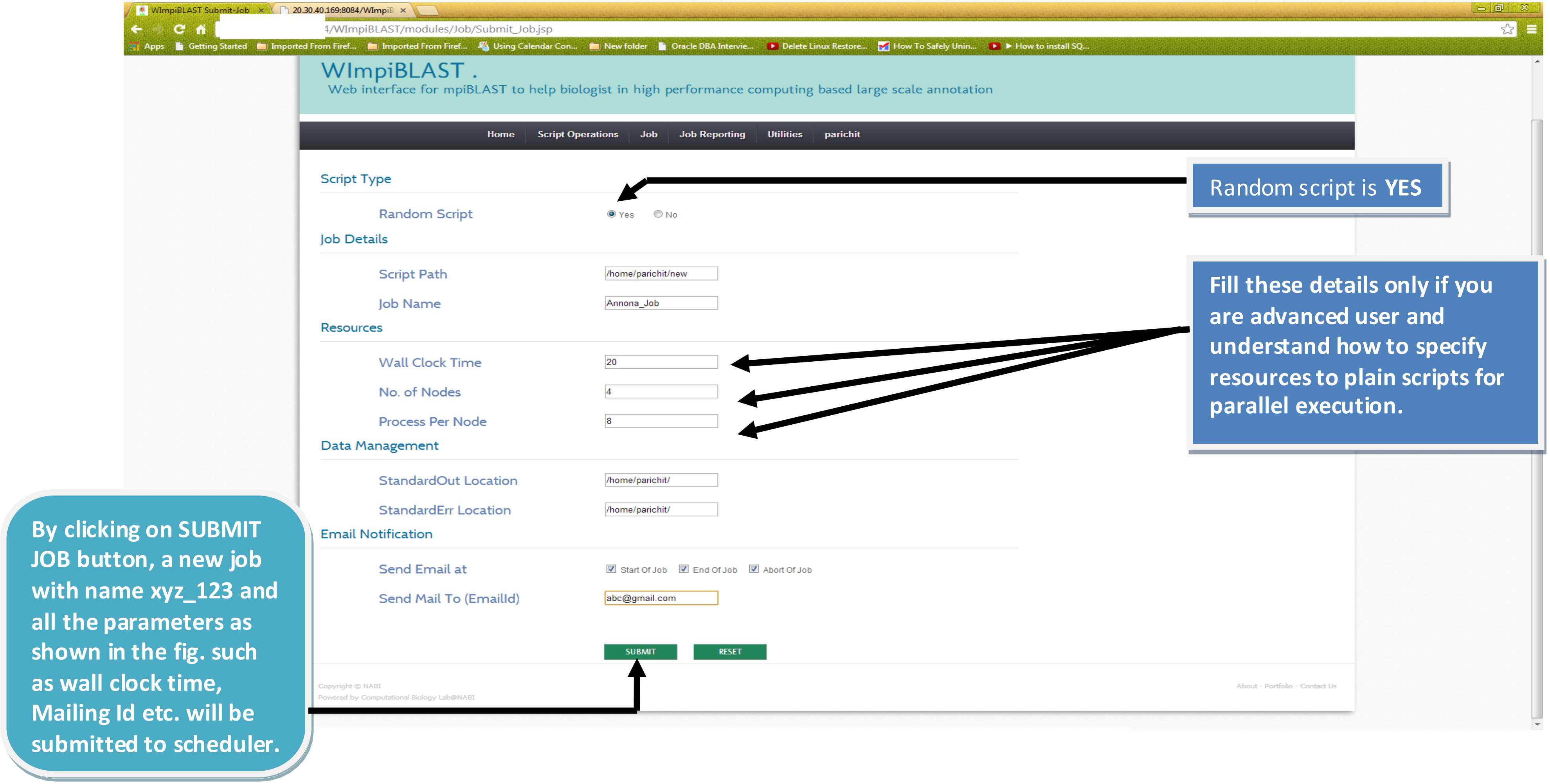

Figure: Random job submissions

Submit job snapshot: Random script: No

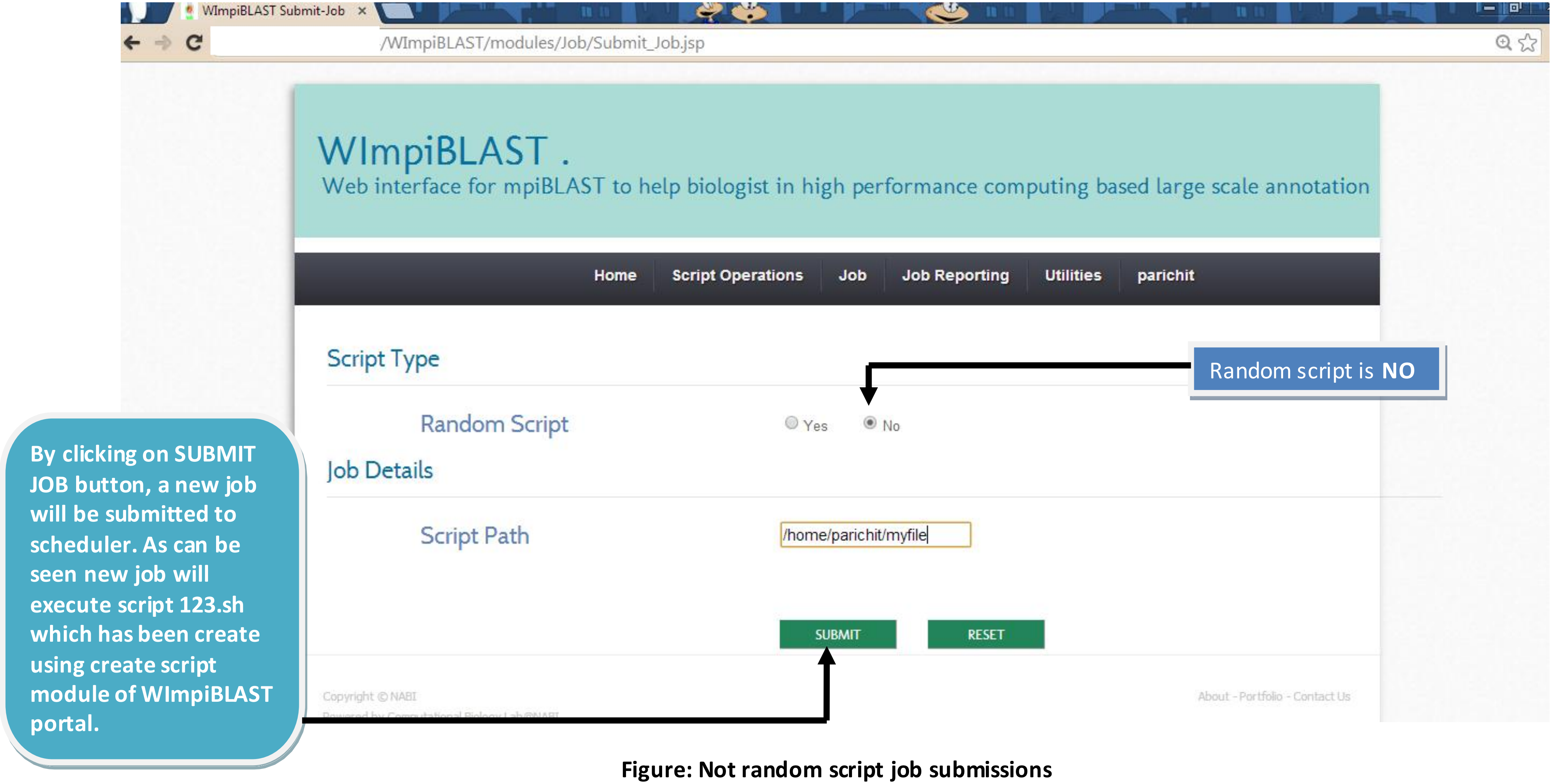

Figure: Not random script job submissions

Submit Script Page Contains / Parameter Information: A brief list of various sections present on this page is as follows:

Job reporting
Script path
Job name

Resources

Number of nodes
Process per node
Wall clock time

Email notification

Notify
Mailing ID

Data Management

Std. output
Std. error

Job script

This parameter specifies the exact location in the file system where the executable script is present. In other words it represents standard input path where the input file is present. If not automatically selected, it can be selected by the user using the path finder functionality of the WImpiBLAST portal.

Job name

Job Name is the name given to the job script with which the particular job is run.

Number of nodes: This parameter specifies the total number of compute nodes that will be used for simultaneously executing the script.

Process per node: This parameter specifies the total number of processes which will be simultaneously executed per node for execution of script.

Wall clock time: This parameter specifies the total amount of time allocated to the script for execution. For e.g. if the script execution crosses this allotted time interval than job scheduler will automatically suspend script execution and an email will be sent to the recipient email address.

Email notification

Email Notification section contains the events when the user is notified by electronic mail to the mail addresses specified to the mail Ids specified.

This electronic notification enables the user to monitor the status of its submitted jobs; by specifying flags we can get mails during any job suspension, completion, abortion etc as per the job status.

Standard output file location

The standard output redirection can be used to write the output to another place, the standard output field consists of a text box. If the specified path is a directory the output streams are put with the default names under that directory. Please note that the standard output file is always created by job scheduler and it is separate from normal output file created by application. Even if user does not specify any output file name then also a standard output file will be created by job scheduler to record successful completion of job.

Standard error file location

The standard error redirection can be used to write the output to another place, the standard error field consists of a text box. If the specified path is a directory the error streams are put with the default names under that directory. Please note that the standard error file is always created by job scheduler and it is separate from normal error created by application. Even if user does not specify any error file name then also a standard error file will be created by job scheduler to record errors thrown by application during execution.

3.3 Job Reporting

The Job reporting feature of WImpiBLAST portal keeps the user up to date about status of various submitted jobs in the system. Generally, a job can be in **queue**, **running**, **halt** or hold and **released** states. Each of these states has its own significance in the life cycle of the submitted job. By default a user can only view jobs to which the user is authorized to view in other words, jobs submitted by another user will not be displayed to the current user. A generic view of current job status in the system will be displayed to the user when user selects the **view job** module. The Job reporting module is designed with a perspective of keeping complexity as minimum as possible while displaying the job status in most user friendly manner to the end user as possible.

User can dynamically hold or release any job by using the control panel present on the right side of screen, thus user has to be concerned only with the end results and not with the exact implementation of procedures.

View job module is helpful in pin pointing the selected job for further detailing to the end user as any selected job will be displayed with all its relevant parameters using which it was created. However, only those jobs which are in queue can be changed or modified by the end user because resource managers or job schedulers does not allow modification of running jobs or released jobs by convention.

3.3.1 Job Monitoring & Management: The functionality provided by Job reporting module can be roughly divided into monitoring and management modules. Whereas, monitoring modules are designed primarily for job monitoring purpose by reporting and displaying the current job status in the system to end user.

The management functionality is intended to change job status as per user requirement. A separate control panel for changing the job status is provided on the right side of the screen using which the jobs can be **hold**, **released** or even **deleted** by user intervention.

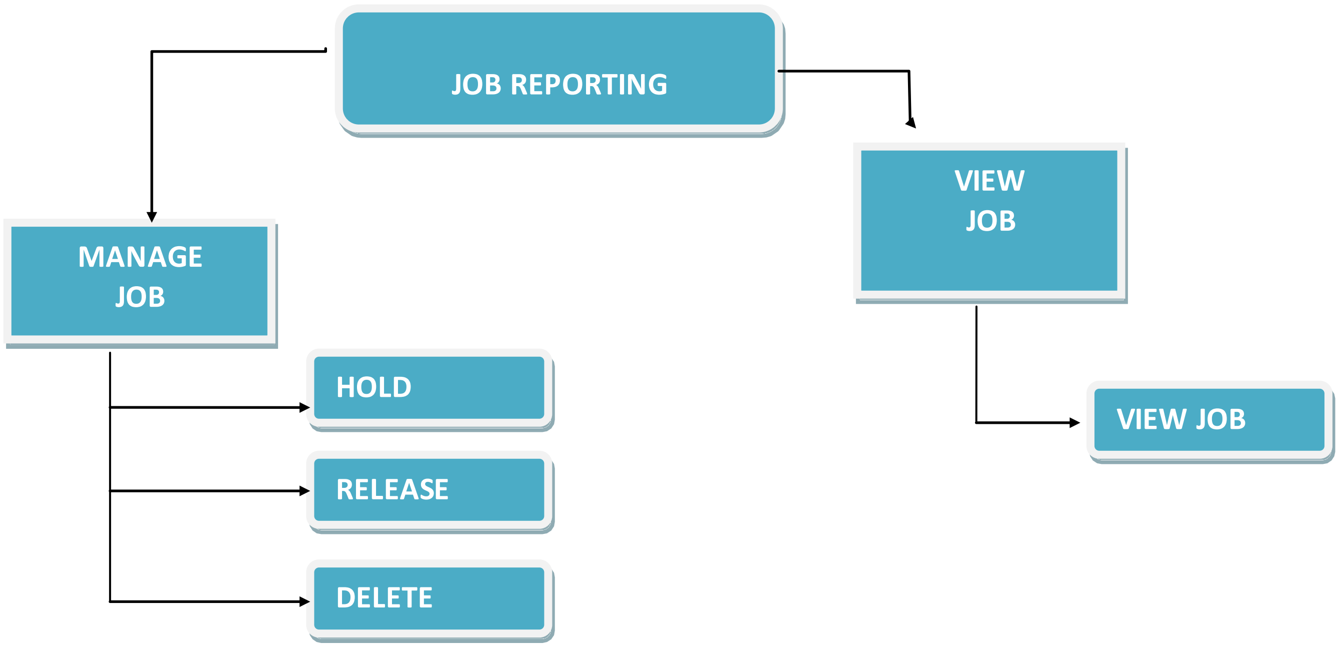

Figure: Job management and monitoring components

WImpiBLAST Portal provides user with an easy monitoring and managing utility for their submitted jobs. It provides information about the current status of the submitted job, for convenience the portal has the facility to watch job specific to a particular status, which makes the things lot clearer.

User can manage these jobs with ease. Portal display information such as: JobID, Priority, JobName, Owner, Status and Queue. User can manipulate selected job for Delete, Hold, and Release.

3.3.1 Job monitoring – View job snapshot:

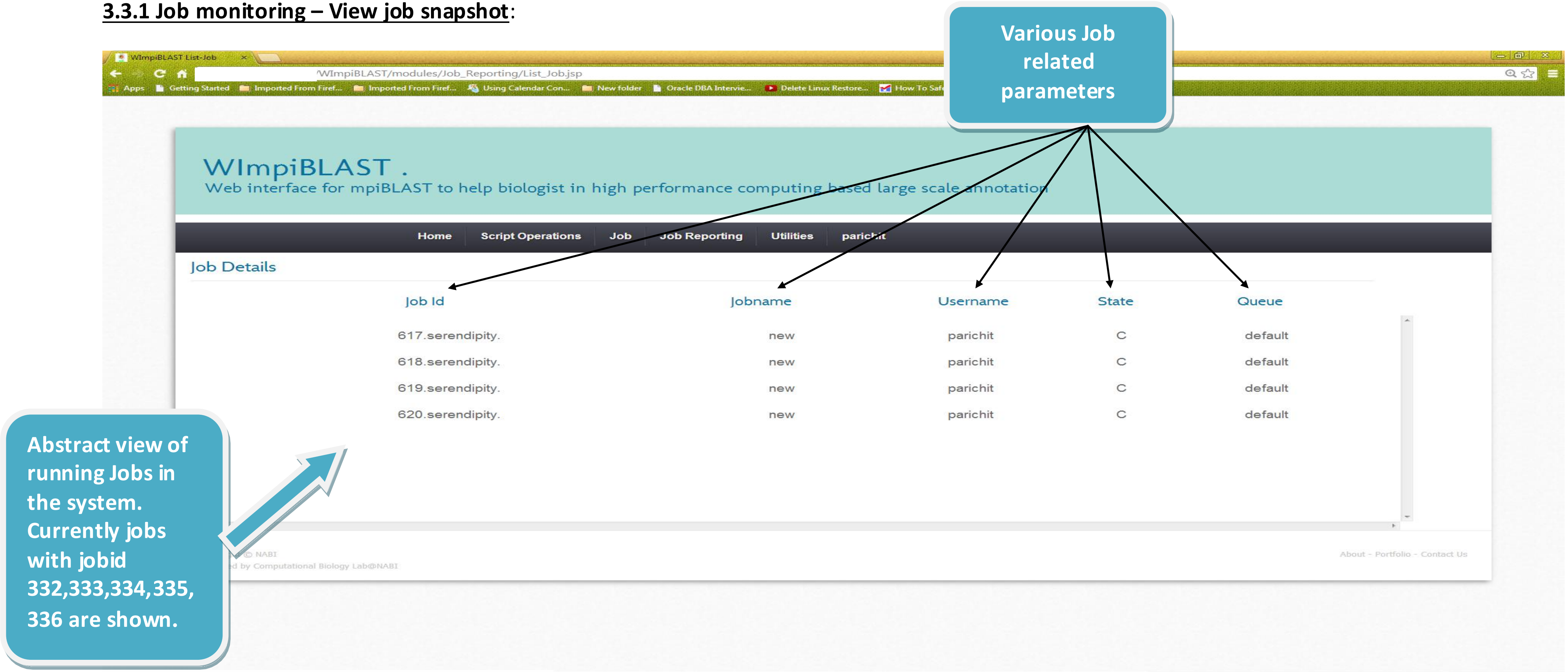

Figure: Job monitoring: View job

Above figure clearly displays a generic snapshot of the system image of running jobs. Job with jobid 617 is completed indicated by symbol “C” in column named ‘state’, completed means that the job is execution is either successful or unsuccessful. User has to refer to error and output files to verify whether job was successful or not. List job provide the user an abstracted view of the jobs in the system.

3.4 Job management:

The Job management module of WImpiBLAST portal provides the flexibility of changing the job status dynamically online. The user can change the status of jobs submitted to the scheduler by making use of control panel (refer fig. 3.1.3.2) on the right side of the screen that contains different status altering options. Whenever, user wants to use the job management functionality, user has to select any one job out of the listed jobs which are displayed in the "view job" module, after job has been selected then user can modify the status of that job by using the options given on control panel on the right side of the screen .The job management module is sub divided into following functions which are as follows:

- DELETE
- HOLD
- RELEASE

DELETE: This option will delete the job selected by the user from the queue. Once deleted that job cannot be resubmitted again for execution. If the user wants to submit a job again with the same parameters as the deleted job , then a fresh job has to be submitted using the submit script option (please refer Submit Script in Job Submission).

HOLD: this option will put the selected job on "HOLD", in other words the status of job will change from "R" to "H" if the job is hold. During" HOLD", that job will temporarily suspend its execution unless user releases the job again by clicking on "RELEASE" option in the control panel. After being released the job will resume its execution as normal with all the parameters as originally being specified by the user when submitting that job.

RELEASE: This option will release the selected job from "HOLD" and the scheduler will restart execution of that job from where it was hold.

3.4 Job management – Manage job snapshot

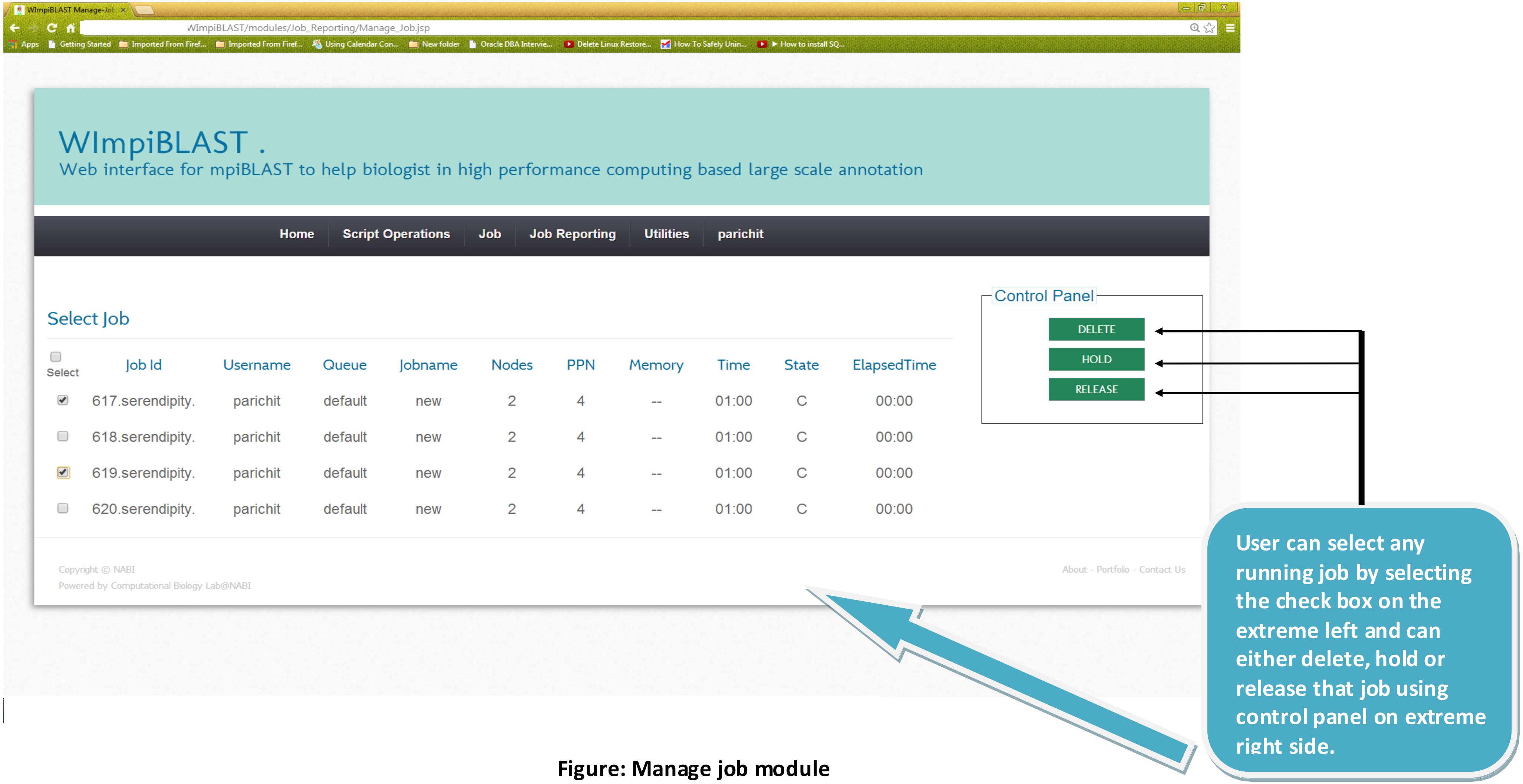

Figure: Manage job module

3.5 Utility: File Manager

The file manager module of WImpiBLAST portal provides the functionality of online downloading of remote files or directories to the client system through tree view facility of WImpiBLAST. This module helps user in managing their files and folders any time between their remote cluster and client desktops, laptops etc. User only needs to select the specific file or folder from their home account on remote cluster to download the file or folders in to their personal computers. This module makes it easy for users to manage file transfers between cluster and their personal computers. The File Manager module can be sub divide into following modules:

- Download utility
- Upload utility
- Tree view utility
- View script utility
- See inside files utility
- Path finder utility

3.5 Utility: File manager snapshot

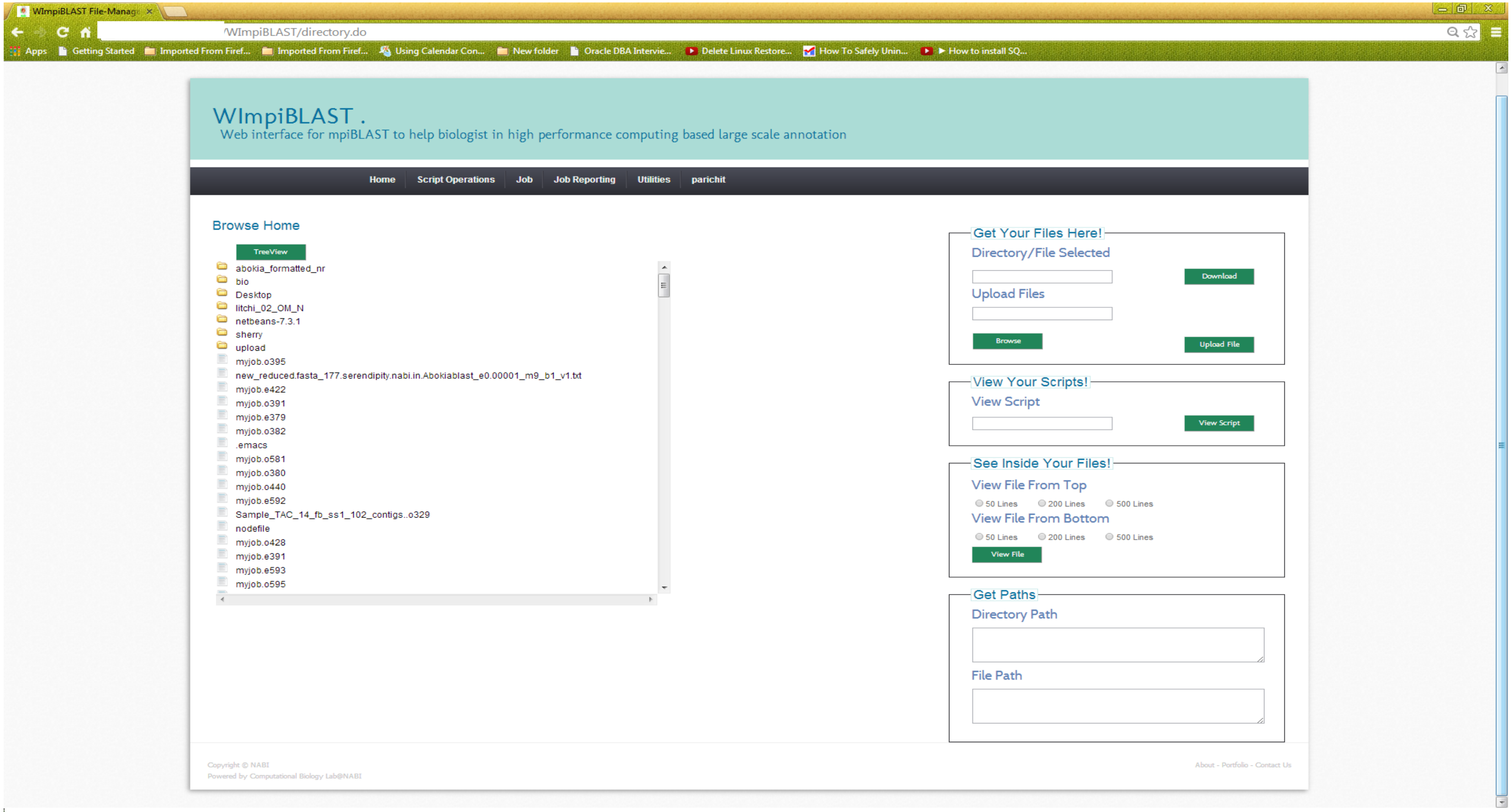

Figure: File manager

3.5.1 (A) Download utility:

In download Utility, user has to select a particular file or directory on the remote system using the “Tree View” utility of WImpiBLAST portal, after user has selected the file or folder download can be started by clicking on Download button. By default all the downloaded files or directories will be placed in to the download folder of the client system. Currently multiple files or folders cannot be downloaded by user but at a time user can download only single file or folder.

3.5.1 (B) Upload utility:

In upload module, user has to select a file from his/her system, after the file is selected, it can be uploaded to server by clicking on Upload button. The user can only upload files but not directories because of security vulnerabilities pertaining to transfer of file system information over the internet, directory uploading has been disabled. However if extremely necessary then the required directory has to be converted into zip (compressed) file format or any other compressed file format and then the compressed file can be uploaded normally by using the upload module functionality. All the uploaded files will be uploaded to “Upload” folder inside home directory of the user by default.

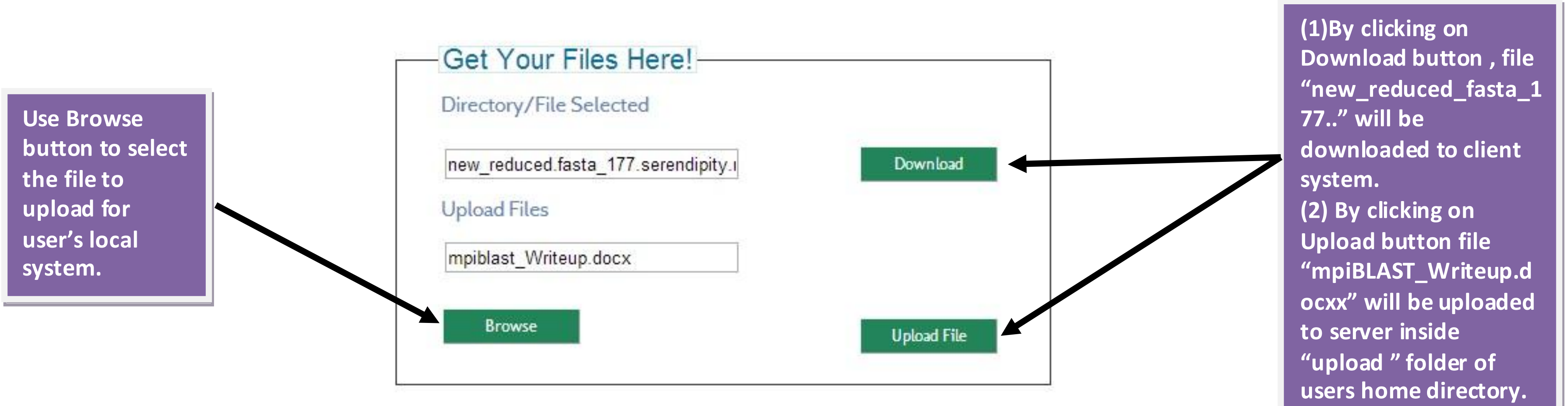

Figure: Download /Upload utility of file manager

3.5.3 Tree view utility:

The tree view utility presents a generic tree view for the home directory of the logged user. All the directories and files in the home directory of the user are represented in hierarchical format when user clicks on ”Tree View Button” located on left side of File Manager module. User can browse through the directories to further extend or collapse the directories. This module is ideal in case the user wants to quickly find the contents of any particular directory or if the user wants to locate any particular file or folder within the home directory. In case the contents of home directory overflow the window, user can use the scroll bar locate on both right side and bottom of tree view window to further browse through the directories and files.

<u>3.4.1.2 Tree View utility snapshot</u>

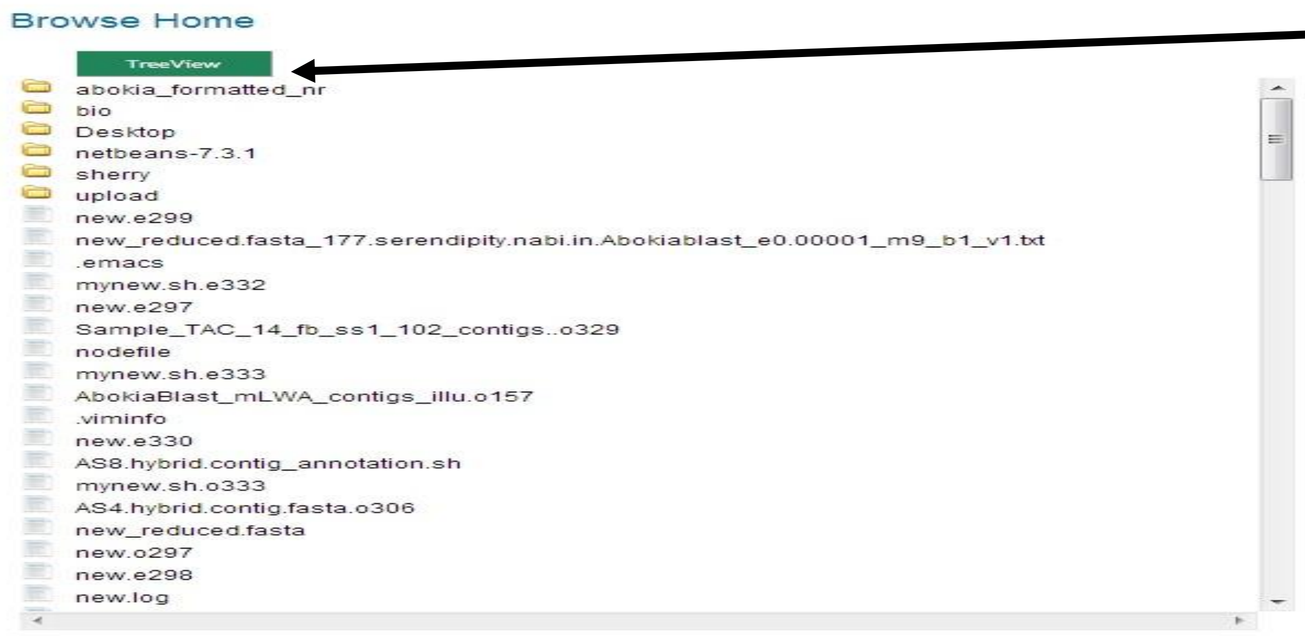

By clicking on “Tree View” button all the contents of user’s home directory will be displayed as shown in this snapshot.

Figure: Tree view utility of file manager

3.5.4 View script utility: The View script utility allows viewing the contents of scripts created by the user. View script can be used make sure that the script created by the user is properly created. The user can also use this utility to manually copy/paste the contents of the script to be used in another script. Upon clicking on "View Script" button the selected script will be opened in new browser window in read only mode thus the user can easily copy/paste the contents of the script. Please note that there are size related limitations on the script that can be viewed in the browser. Currently user can view only scripts up to 1mb of size in browser.

View Script button will open the "new_reduced_fasta_177…" script in new browser window .

<u>3.5.4 View script utility snapshot</u>

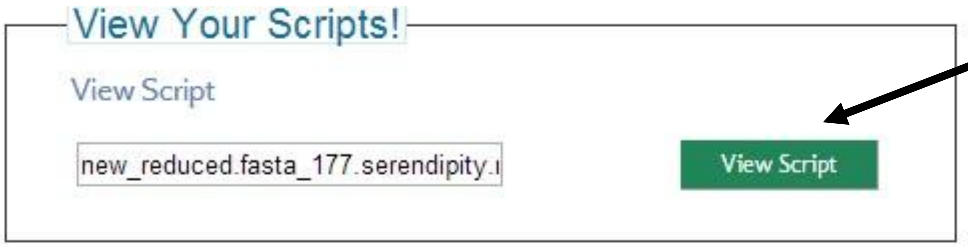

Figure: View script utility

3.5.5 See inside files utility: This utility has its importance when script/file that user wants to view is larger than 1mb in size. For e.g. user wants to view the contents of some file that is larger than 1mb so that file can't be opened using "View Script" utility. In "See inside Files" utility is subdivided into two categories as follows:

- **View from top:** View the file/script from top have possible options as top 50 lines, top 200 lines and top 500 lines**.** User can select any of the criteria after selecting the file from left "Tree View Module" to view the file in new browser window.
- **View from bottom:** View the file/script from bottom have possible options as bottom 50 lines, bottom 200 lines and bottom 500 lines**.** User can select any of the criteria after selecting the file from left "Tree View Module" to view the file in new browser window.

One possible condition where user might want to use this utility is when there is some long running job executing in the system. Since such long running jobs will take considerable period of time to complete hence there is no single way to trace their progress. “See Inside Files” utility will allow the user to view selective portions of output file being written by the executing job so user can track that how much of that file has been written or how much of input has been processed.

By viewing the bottom 50/200/500 lines of the output file, user can ascertain the progress made by executing job or how much input data has been processed.

3.5.5 See inside files utility snapshot

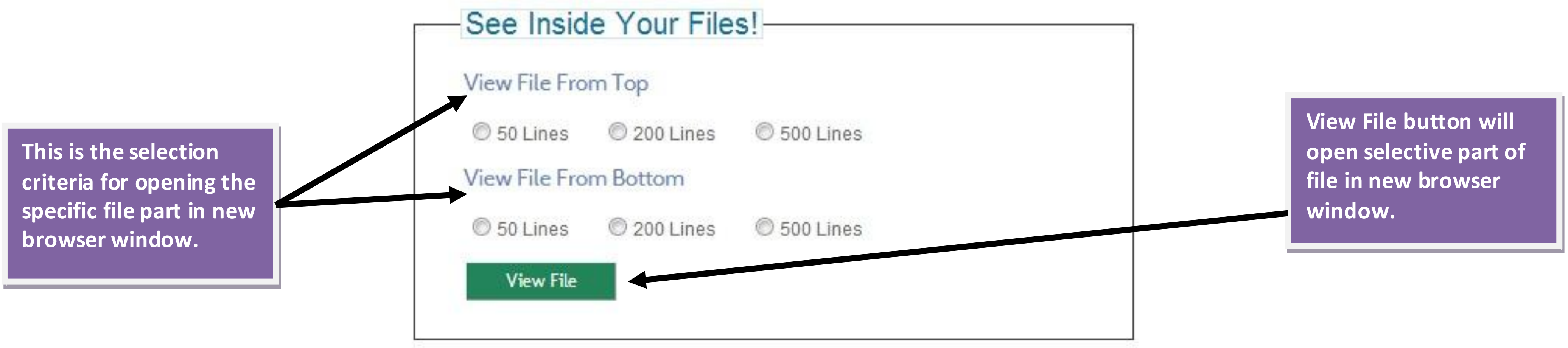

Figure: See inside your files utility

3.4.1.5 Path information utility: Get Paths utility is useful in every other module of WImpiBLAST portal. Get Paths utility dynamically generate paths to files and folders in the system. For e.g. if user wants to create the script at particular location in the system then the path to that location is required in the create script module. By selecting the specific folder/directory from the "Tree View" module on left side of file manager utility , the paths to directory are automatically generated in the Directory Paths column of "Get Paths" utility. User can copy and paste that path in create script module to create script at that location only.

Likewise, by clicking on any file in Tree View utility, the path to that file is generated in the File Path column of "Get Paths" utility. For e.g. there is some script that user wants to modify so user just need to click on that script in "Tree View Module" to generate path to that script. Once the path is generated the user can copy and paste that path in Modify Script module to open that script for modification.

<u>3.4.1.5 Get paths utility snapshot</u>

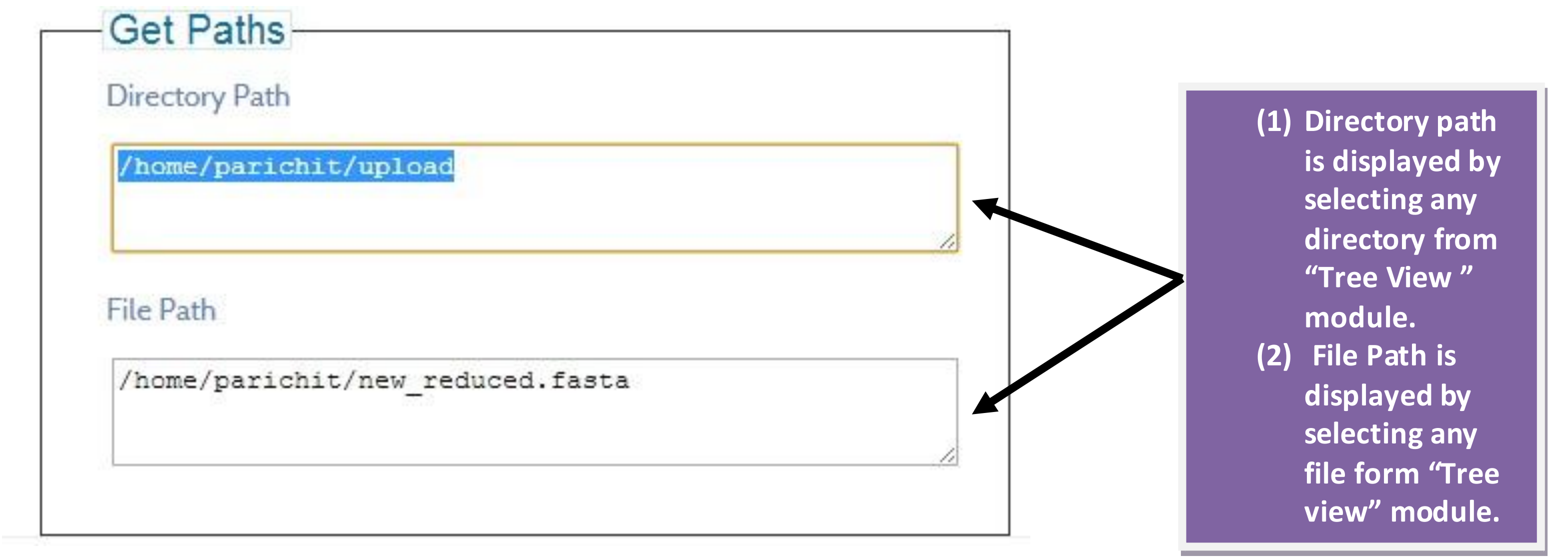

Figure: Get paths utility

3.6 Manual:

This module will display the user manual to the end user for reading. The user manual is displayed in pdf (portable document format) format so that any user can either read it online or can download it for reading later. The manual is in pdf format, hence can be opened with ay pdf reader and it will displayed the same irrespective of software used for reading the pdf. The user manual is an important documentation about various features and functionalities of WImpiBLAST portal. It highlights major advantages of WImpiBLAST portal and also describes in detail the objective and usage of each and every module of the portal.

For Downloading the User manual: User has to click on Manual menu item to open the manual in pdf format. Once the browser displayed the manual, user can simply download it by right clicking on the page and selecting option "**Save Page as**".

3.7 Miscellaneous utilities: WImpiBLAST portal also consist of additional utilities that quantify the primary modules of the portal for achieving proper functionality in those modules. These are as follows:

- Input Validation Functionality
- File Size Validation Functionality
- Path Finder Functionality

3.7.1 Input validation functionality:

This feature is responsible for alerting the user about empty fields that are necessary for a particular module. While this feature appears to be so trivial but it is responsible for alerting the user about possible empty information fields and required parameters of a module.For e.g. while creating script if user left any mandatory field then this feature will automatically alert the user about required information and will prohibit the user from progressing or completing the module until unless all mandatory fields are filled.

Please note that this feature is not responsible for checking the input files uploaded by user or for that matter any i/p, o/p files. User has to make sure that uploaded files should be in proper format as required by the blast application program.

3.7.1 Input validation feature snapshot

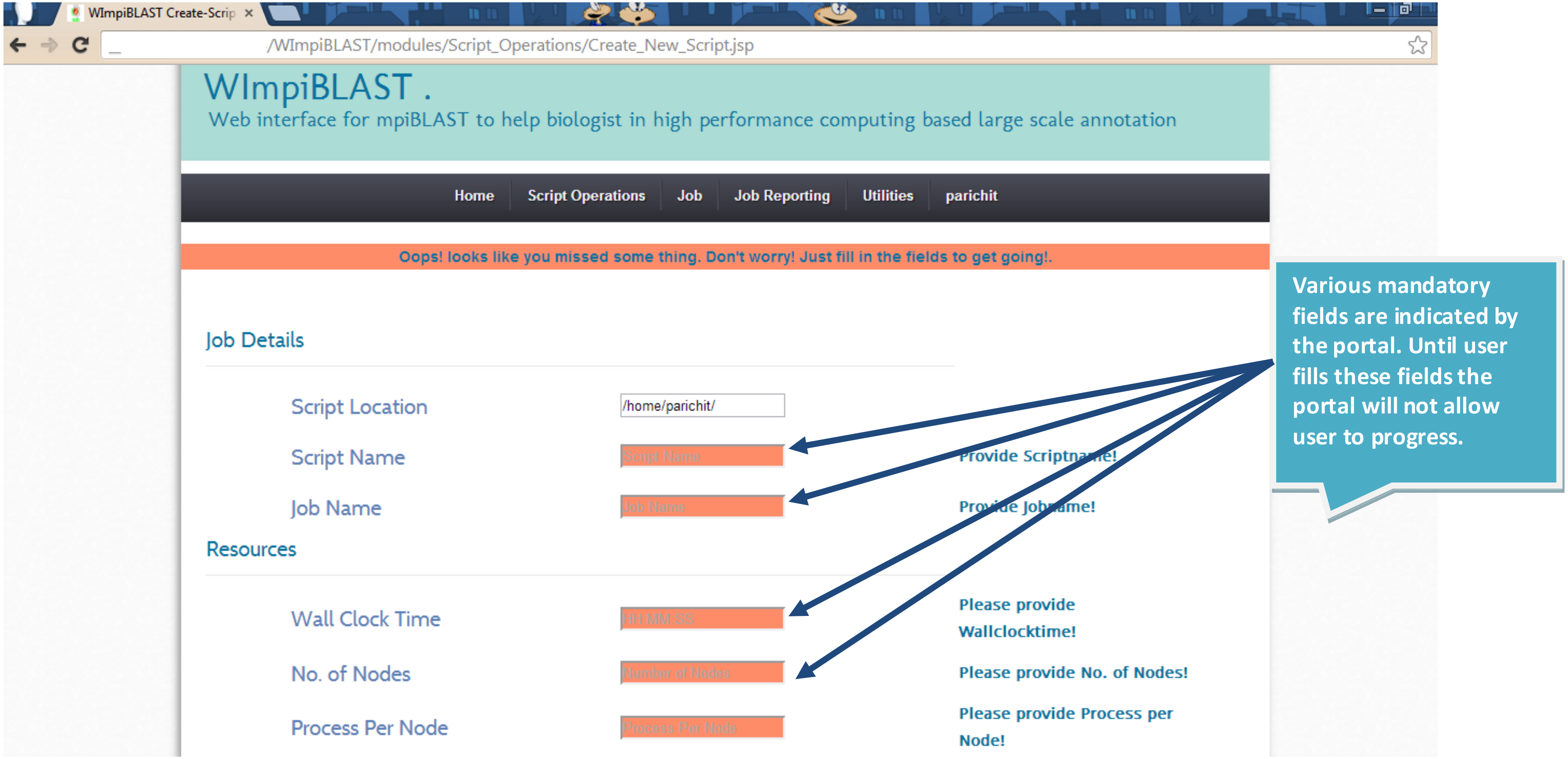

Figure: Input validation feature

3.7.2 File size validation:

This functionality is one of the several safety standards implemented in the code of WImpiBLAST portal. Basically this feature restricts the user from violating file size limitations as imposed inside the code. However these file size values can be easily customized inside the code. For an instance currently upload file size cannot exceed 200mb and download file size cannot exceed 100mb at most. If user tries to violate these file size limitations an alert prompt will be displayed acknowledging the user about feasible file size restrictions.

Apart from standard fault tolerance mechanism, this feature also reduces load on network bandwidth by limiting the size of file that can be downloaded/uploaded by the user through browser. Therefore this feature makes the overall code performance more robust while restricting the users from overwhelming the bandwidth and network resources.

<u>File size validation Ssnapshot</u>

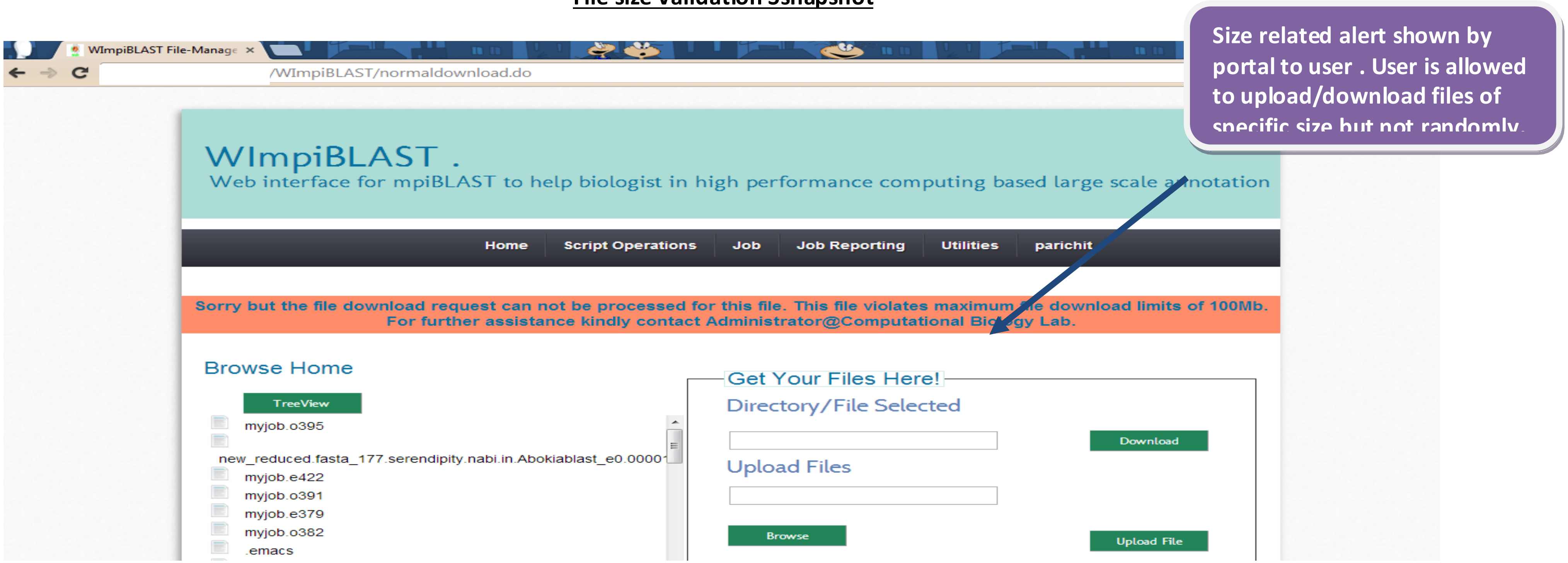

Figure: File size validation alert shown to user

3.7.3 User alert functionality:

User alert feature works in conjunction with above mentioned features. User alert feature is responsible for making end user experience smoother or more comfortable indeed. In case portal encounters some irrecoverable error this feature will inform user about the nature of error along with specific error code. The page that will be displayed will ask the user to note the error code and inform the administrator about what series of actions caused the error. Having this feature resulted into more robust code and better user experience.

Now user no longer has to worry about cryptic error code and warnings thrown by the browser which are simply un-interpreted to common user. User can simply inform the administrator about possible error code or warnings for rectification while still resuming their normal operations on portal.

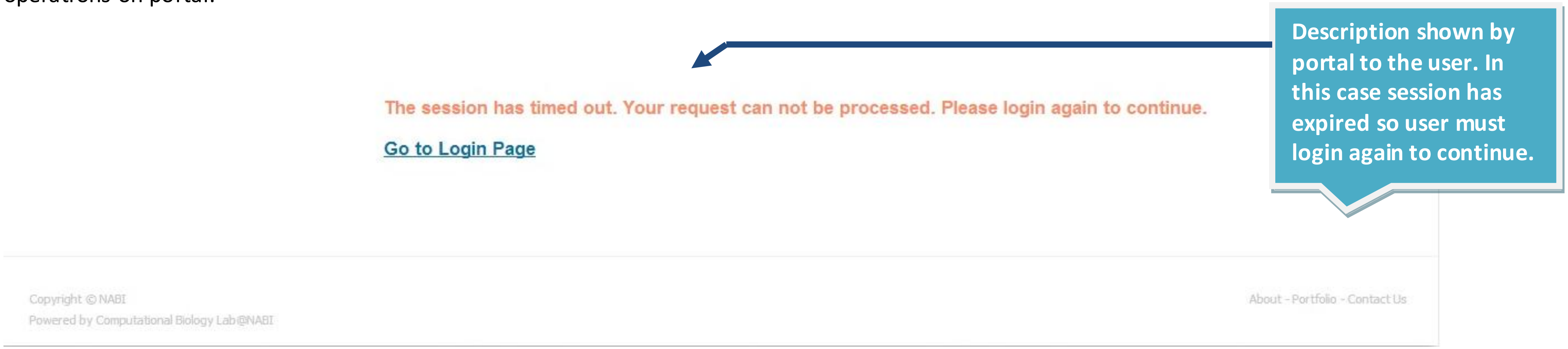

Figure: Error code containing page shown to the user

3.8 Logout

Logout will finally log off the user from WImpiBLAST portal screen and the user will be redirected to the login screen once again. After logout, the user will not be able to view or use any or all the features of WImpiBLAST portal because logging out from portal also terminates the connection to the remote cluster and hence all the session data will be destroyed after logout.

Note: Current version of WImpiBLAST portal supports a session time of 30 minutes hence if user does not log out of portal but somehow accidently closes the window then also user can access the portal without logging in again because session is valid. For accessing the portal again in case window is closed accidently, just type url to access portal in browser navigation bar.

Typically url to access portal is like follows

Ip-address: port number/WImpiBLAST

xxx.xxx.xxx.xxx:8080/WImpiBLAST

Appendix

1. **Deployment server or hosting Server:** This term refers to system on which WImpiBLAST.war file is copied and all the users will access WImpiBLAST by using ip_address of this server in their browser.

2. **Mpirun/Mpiexec path:** Administrator can provide either mpirun path or mpiexec path whichever is present on their system.

   For details see: http://www.mpich.org/

3. **User accessible**: mpirun, mpiexec and mpiblast binaries should be accessible by all the users of the system i.e these binaries should be present at path that is readable and executable by all users but writable by only administrator or root.

4. **Mpi:** Message Passing Interface is a library or collection of routines and functions to enable distributed communication and i/o in multi-core systems or HPC clusters or Supercomputers.

   For details: https://computing.llnl.gov/tutorials/mpi/

5. **HPC:** High Performance Computing refers to parallel computing systems that can used to speed up otherwise intensive calculations.

6. **BLAST:** Basic Local Alignment Search Tool is a Sequence Similarity Search application used for annotation of genome.

   For Details: http://blast.ncbi.nlm.nih.gov/Blast.cgi

7. **Job:** In context of this document only, a job refers to a executable script that is submitted for execution to job scheduler by user.

8. **mpiBLAST:** mpiBLAST is parallel version of NCBI's BLAST application**.**

   For Details: www.mpiblast.org

END OF MANUAL
