# Supplementary material for: WImpiBLAST: Web Interface for mpiBLAST to Help Biologists Perform Large-Scale Annotation Using High Performance Computing
Source: PLoS One. 2014 Jun 30;9(6):e101144. doi: 10.1371/journal.pone.0101144 (PMC4076281; doi:10.1371/journal.pone.0101144)
Supplement: Text S2 — Installation Manual. (PDF) [file pone.0101144.s004.pdf]

NABI

National Agri-Food Biotechnology Institute

WImpiBLAST

Installation Manual

Document Version: 1.0

Product Version: 1.0

2013, National Agri-Food Biotechnology Institute (NABI)
V-1.0

About Document

This document briefly describes the minimum requirements and installation process for WImpiBLAST web portal on the cluster. This is not a comprehensive document and is aimed at providing indicatory information about installation of WImpiBLAST on cluster.

In case of any doubt or unresolved issue that is not covered in this document please send an email *to* ***ict@nabi.res.in***

This manual is expected to be used strictly by system administrator or superuser i.e. 'root' user because most of the requirements mentioned in this manual requires superuser privileges to be implemented.

INDEX

About WImpiBLAST.. 4

Minimum Requirements.. 5-6

Software and Dependencies... 5

Basic Hardware Requirements... 6

Configuration.. 6

Installation Process... 7-8

Appendix.. 9

About WImpiBLAST

WIMPIBLAST Portal is a user-friendly parallel application submission, management and monitoring interface that use open source Job scheduler and Resource Managers i.e. Torque. NABI's Computational Biology Lab has developed WImpiBLAST, a web UI developed specifically for mpiBLAST. It is developed to give a user-friendly interface for molecular biologist to submit mpiBLAST job on HPC cluster.

Routinely most of the molecular biologist use NCBI portal for doing sequence similarity search for their gene of interest. With the advent of Next Generation Sequencing Technologies it has now become possible to study gene expression at Genome wide scale through RNA-seq experiments. Annotation of all the genes in a given transcriptome is computationally very intensive job which can be accelerated by using High Performance Computing cluster.

To do this transcriptome annotation themselves, Molecular Biologist are often limited due to steep learning curve to gain skills of programming and command line usage. By developing WImpiBLAST we are trying to help molecular biologist to overcome this limitation by helping them use high performance computing cluster for computationally intensive annotation jobs through a simple web interface.

Minimum Requirements

Following sections illustrates about minimum requirements for installing WImpiBLAST web portal on HPC cluster. Most of the software dependencies are already a part of most Linux distributions so you just need to install them if not already installed at the time of cluster setup.

Software and Dependencies: WImpiBLAST requires following applications to be installed on HPC cluster to function properly.

Apache-Tomcat Server -7.x.x: Apache Tomcat is the default application server used by WImpiBLAST. Apache-Tomcat is a necessary requirement for WImpiBLAST to work. Current release of WImpiBLAST has been tested with apache-tomcat-7.0.42 but higher version of apache server should also work without any issues. Please note that apache-tomcat should be installed and run by superuser i.e "root" user to enable full access to required system executable and paths, for more detail please refer to 'Installation Process'. Apache-tomcat is part of most Linux distributions and so it should already be present on your system. In case you are unable to find it or install it please contact your system administrator. For more details refer to
http://tomcat.apache.org/

mpiBLAST-1.6.0: mpiBLAST should be installed and accessible by users on the cluster node on which WImpiBLAST will be deployed. We have tested WImpiBLAST by deploying it on master node but different sites may have different deployment scenarios depending on their requirements. In nutshell make sure that the node on which WImpiBLAST has to be installed has working installation of mpiBLAST that is accessible by all the users having account on the cluster. Current release uses mpiblast-1.6.0 and it is strongly recommended that user should not use older versions as it may result into performance degradation. For more details please refer to
http://www.mpiblast.org/

Torque resource manager (formerly known as PBS/Maui)(3.0.2, 3.0.5): Torque is required to enable job submission/reporting feature of WImpiBLAST. If torque is not installed then user will be able to use most the features of WImpiBLAST except job submission/reporting. It is strongly recommended that user should ask their administrator to install Torque because job reporting and management feature of WImpiBLAST depends on Torque installation. If the cluster is setup using rocks then chances are that torque might already be present. For more details please refer to
http://www.adaptivecomputing.com/products/open-source/torque/

JDK (Java Development Kit): JDK is a necessary component of WImpiBLAST requirements. JDK is part of most Linux distributions and should already be present on your cluster. If the administrator has intentionally prevented JDK installation at the time of cluster setup then you should ask your administrator to setup JDK on cluster and made it accessible by all the users on system. Current release has been tested with java-1.7.0 but higher version should also work without any issues. For more details please refer to

http://www.oracle.com/technetwork/java/javase/downloads/index.html

Hardware Requirements: There are no stringent hardware requirements for installing WImpiBLAST however it is advised that better hardware capacity will add up to run time performance of WImpiBLAST especially in case several users are accessing it simultaneously.

There should be at least 2 GB of RAM available in node on which WImpiBLAST will be installed and 100 MB of free disk space is required in partition in which apache-tomcat server is installed for storing files of WImpiBLAST.

Configuration: WImpiBLAST reads parameter values from its configuration file '.WImpiBLAST.conf' present inside '/etc' directory on server for performing some validation functions in the code.

.WImpiBLAST.conf file can be downloaded along with WImpiBLAST.war file.

It is very easy to configure settings for WImpiBLAST using this file. There are five parameters in this file which needs to be filled before using WImpiBLAST. The parameters are ip_address of server (usually master node of cluster), path to mpirun binary on server, path to mpiblast binary on server, total number of nodes in the cluster and number of cores per node.

This file has to be copied in '/etc' directory of deployment server before user can login through WImpiBLAST in web browser. It is strongly recommended that only administrator should edit this file. Unless parameters in this file are not configured properly WImpiBLAST will prompt user about misconfigured file or absence of any necessary parameters in file. For more details please refer to '**Installation process**' section.

***Note**: Please note that this section only list direct dependencies for running WImpiBLAST, if there are any secondary dependencies required on your specific cluster then those lies outside the scope of this document. For e.g. if the cluster does not have working installation of mpiBLAST then installing and using it requires to first install MPI library on cluster which is a secondary dependency. In case of any such doubts or issues please do not hesitate to contact us at **ict@nabi.res.in**.*

*We strongly recommend using equivalent or higher version of above mentioned software's and dependencies not older versions. If currently older version are installed then kindly ask your administrator to update them accordingly. In case of any such doubts or issues please do not hesitate to contact us at **ict@nabi.res.in**.*

Installation Process

Installing WImpiBLAST is matter of few steps once all the software requirements are fulfilled. Installation need three steps to be followed in respective order as described below. Administrator is expected to follows these steps as they are listed here and not in random manner.

Step 1: Setting up WImpiBLAST.war file: Download the WImpiBLAST.war file form *www.nabi.res.in* and copy this file in 'webroot' of apache-tomcat server. If you do not understand what webroot means then just copy the file in 'webapps' directory inside apache-tomcat installation directory. By copying the WImpiBLAST.war file inside webapps directory it become accessible to users when they access following URL in their web browser.

URL-1: x.x.x.x:8080/WImpiBLAST URL-2:x.x.x.x:8084/WImpiBLAST

Note: *Please note that x.x.x.x indicates the ip-address of server on which WImpiBLAST.war was copied or the deployment server. The two URL indicate the specific port on which apache service is running. By default for most implementations it is port number 8080 but in some cases it can also be port 8084. Please note that administrator can setup apache-tomcat to use any port depending on requirements and system policies, if that is the case then please replace 8080/8084 in above URL by that port.*

Step 2: Edit permissions in default apache-tomcat policy file*:* By default apache server can't access the home directories of users on server so the default 'catalina.policy' file needs to be edited to modify access permission for apache server. 'catalina.policy' file can be found inside 'conf' directory of apache-tomcat installation folder.

Caution: Please make a copy of catalina.policy file in case something goes wrong and rename the copy as catalina.policy_backup. In case you ever messed up with your catalina.policy file you can always rename the backup file to Catalina.policy and resume normally.

Now open original catalina.policy file in editor of your choice and scroll to bottom of the file, paste following lines there and leave everything else unchanged,

```
grant codeBase "/home/" {

  permission java.io.FilePermission "read,write,execute"

 };

grant codeBase "/etc/" {

  permission java.io.FilePermission "read"

 };
```

Now save the file and exit.

Mandatory Test: Restart apache server and try to access WImpiBLAST in web browser, you should see Login screen in your browser. If login screen is displayed without any errors it means that setup is complete and you just need to do one more step i.e. edit .WImpiBLAST.conf file

The configuration file can be downloaded along with WImpiBLAST.war file. Copy the .WImpiBLAST.conf file in /etc directory of hosting server and follow below steps.

Step 3: Edit .WImpiBLAST.conf file: Only 'root' can edit configuration file for WImpiBLAST as these settings are universal and will be applicable for all the users of the system. Administrator is required to fill following parameters in this file.

hostip=*"enter ip address of server that contains WImpiBLAST.war file"*

mpirun_path*="enter path where mpirun binary resides in server"*

mpiblast_path*="enter path where mpiblast binary resides in server"*

All the information has to be entered within "" (double quotes) and there should be no space between = and starting of "".

Congratulations!! If you have performed all the mentioned steps in order you should now have a working installation of WImpiBLAST portal. You are ready to initiate your first job through WImpiBLAST.

Please refer to Quick Start Guide to get started with WImpiBLAST.

Appendix

1. **Deployment Server or Hosting Server:** This term refers to system on which WImpiBLAST.war file is copied and all the users will access WImpiBLAST by using ipaddress of this server in their browser.

2. **mpirun/mpiexec path:** Administrator can provide either mpirun path or mpiexec path whichever is present on their system.

   For details see: http://www.mpich.org/

3. **User Accessible**: mpirun, mpiexec and mpiblast binaries should be accessible by all the users of the system i.e these binaries should be present at path that is readable and executable by all users but writable by only administrator or root.

4. **Mpi:** Message Passing Interface is a library or collection of routines and functions to enable distributed communication and i/o in multi-core systems or HPC clusters or Supercomputers.

   For details: https://computing.llnl.gov/tutorials/mpi/

5. **HPC:** High Performance Computing refers to powerful computing systems that can used to speed up otherwise intensive calculations.

6. **BLAST:** Basic Local Alignment Search Tool is a Sequence Similarity Search application used for annotation of genome.

   For Details: http://blast.ncbi.nlm.nih.gov/Blast.cgi

7. **Job:** In context of this document only, a job refers to a executable script that is submitted for execution to job scheduler by user.

8. **mpiBLAST:** mpiBLAST is parallel version of NCBI's BLAST application.

   For Details: www.mpiblast.org

9. **EasyBuild Tool:** EasyBuild tool can also be used to install mpiBLAST and its dependencies on cluster rather than building from source code.

   For Details: http://hpcugent.github.io/easybuild/

10. **DRMAA Support:** DRMAA can be used to support a variety of resource managers other then torque for job submission and management.

    For Details: http://www.drmaa.org/implementations.php

Notes: Dear user, we have tried our best to help administrator and users in setting up and using WImpiBLAST but if you think there need be improvements or if you face bugs please feel free to report any issue or bug at ict@nabi.res.in or use https://groups.google.com/forum/#!forum/wimpiblast-user-group to ask your questions.

We are a small team of computational biologists and computer science guys who sincerely want to contribute to field of computational biology by helping you out. In case we omitted something or do not incorporate some critical feature that you think can make difference then kindly let us know.

We will try our best to sort you out.
