# Supplementary material for: WImpiBLAST: Web Interface for mpiBLAST to Help Biologists Perform Large-Scale Annotation Using High Performance Computing
Source: PLoS One. 2014 Jun 30;9(6):e101144. doi: 10.1371/journal.pone.0101144 (PMC4076281; doi:10.1371/journal.pone.0101144)
Supplement: Text S3 — Quick Start Guide. (PDF) [file pone.0101144.s005.pdf]

NABI

National Agri-Food Biotechnology Institute

WImpiBLAST

Quick Start Guide

Document Version: 1.0

Product Version: 1.0

2013, National Agri-Food Biotechnology Institute (NABI)
V-1.0

About Document

This document is a guide for using different modules of WImpiBLAST portal. It will serve to teach users about how to get started with WImpiBLAST quickly.

In any way it will not serve the purpose of a detailed manual and does not contain in-depth description about individual modules of WImpiBLAST and their objective. A comprehensive description of individual modules in WImpiBLAST, functions served by them and meaning of different parameters present in those modules can be obtained from WImpiBLAST user manual which serves the purpose of detailed manual.

This guide is not meant for users who want to understand the technical aspects of WImpiBLAST and details of components involved in WImpiBLAST. It is only meant for users to initiate specific tasks in WImpiBLAST or learn usage of specific modules of WImpiBLAST.

It can be used by person who wants to use WImpiBLAST irrespective of previous computational or bioinformatics application exposure. This will help core biologists who want to use mpiBLAST (parallelised version of NCBI blast) to speed up their research and obtain results faster with comparative accuracy to NCBI blast without mandating the knowledge of all command line parameters or parallel script creation.

In case of any doubt or unresolved issue that is not covered in this document please send an email *to* ***ict@nabi.res.in***

INDEX

About WImpiBLAST.. 4

New Features

How to do!

How to execute mpiBLAST Job... 5-6

How to change previous scripts...7

How to submit script after modifying...8

How to monitor running scripts...9

How to manage running scripts...10-11

How to manage files in WImpiBLAST...12-16

How to logout..17

Appendix...18

About WImpiBLAST

WIMPIBLAST Portal is a user-friendly job submission, management and monitoring interface that is compatible with open source Job scheduler and Resource Managers i.e. Torque. NABI's Computational Biology Lab have developed WImpiBLAST, a web UI developed specifically for mpiBLAST. It is developed to give a user-friendly interface for molecular biologist to submit BLAST job on HPC cluster.

Routinely most of the molecular biologist use NCBI portal for doing sequence similarity search for their gene of interest. With the advent of Next Generation Sequencing Technologies it has now become possible to study gene expression at Genome wide scale through RNA-seq experiments. Annotation of all the genes in a given transcriptome is computationally very intensive job which can be accelerated by using High Performance Computing cluster.

To do this transcriptome annotation themselves, Molecular Biologist are often limited due to steep learning curve to gain skills of programming and command line usage. By developing WImpiBLAST we are trying to help molecular biologist to overcome this limitation by helping them use high performance computing cluster for computationally intensive annotation jobs through a simple web interface.

New Features

Following new features have been added in WImpiBLAST.

1. **New Turbo job feature**: Turbo Submit reduces the script creation and submit time cycle manifold, now users only need to browse for their input query file and Click on 'Turbo Submit'. Explore 'Turbo Submit (beta)'.
2. **Experience the Organized Job Directory Structure:** Now, with each successful job, a dedicated directory is created for holding all the files for that job. The directory bears the same name as jobname given by user, to help in remembering the particular files. The jobs directories are created inside 'WIMPIHome' directory of user's home.
3. **Find Script name Information of Jobs:** The script associated with each job is now displayed in "View Job". Helps you to remember which scripts are associated with the running jobs.
4. **Explore New Way of Browsing Files/Folders:** All the files are now sorted by last modification time, so you don't have to look for newly created files every time you submit a job. You will find new files/folders at the top of Tree View. Additionally size of each file is now displayed besides the file to help in assessing the progress of result. Explore utilities --> File Manager --> Tree View.
5. **Create & Download Folder:** Now you can create your own folder to house different files or data at one place. Also, just click on any folder to download it in compressed form. Explore Utilities --> File manager --> Tree View --> Create Folder.
6. **Find Workflow Diagram on Every Page:** A workflow diagram to let you understand how to submit scripts.

How to do!

This section describes briefly about how user can quickly execute their first mpiBLAST job on HPC (High Performance Computing) cluster.

How to execute mpiBLAST Job: To execute mpiBLAST job through WImpiBLAST follow below steps in exact manner as they are given here but not randomly.

1. Go to Script operations tab and select **'Create & Submit Script'** option.

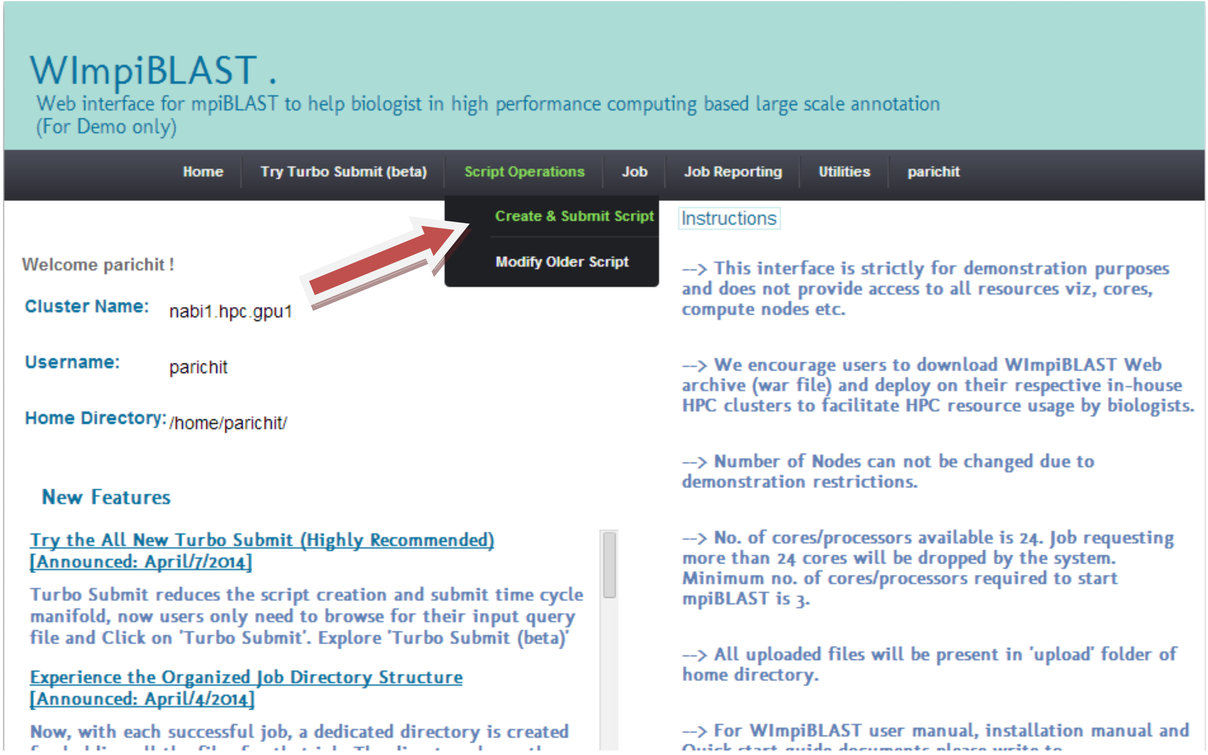

2. By clicking on '**Create & Submit Script'** following page will open. Enter required information.

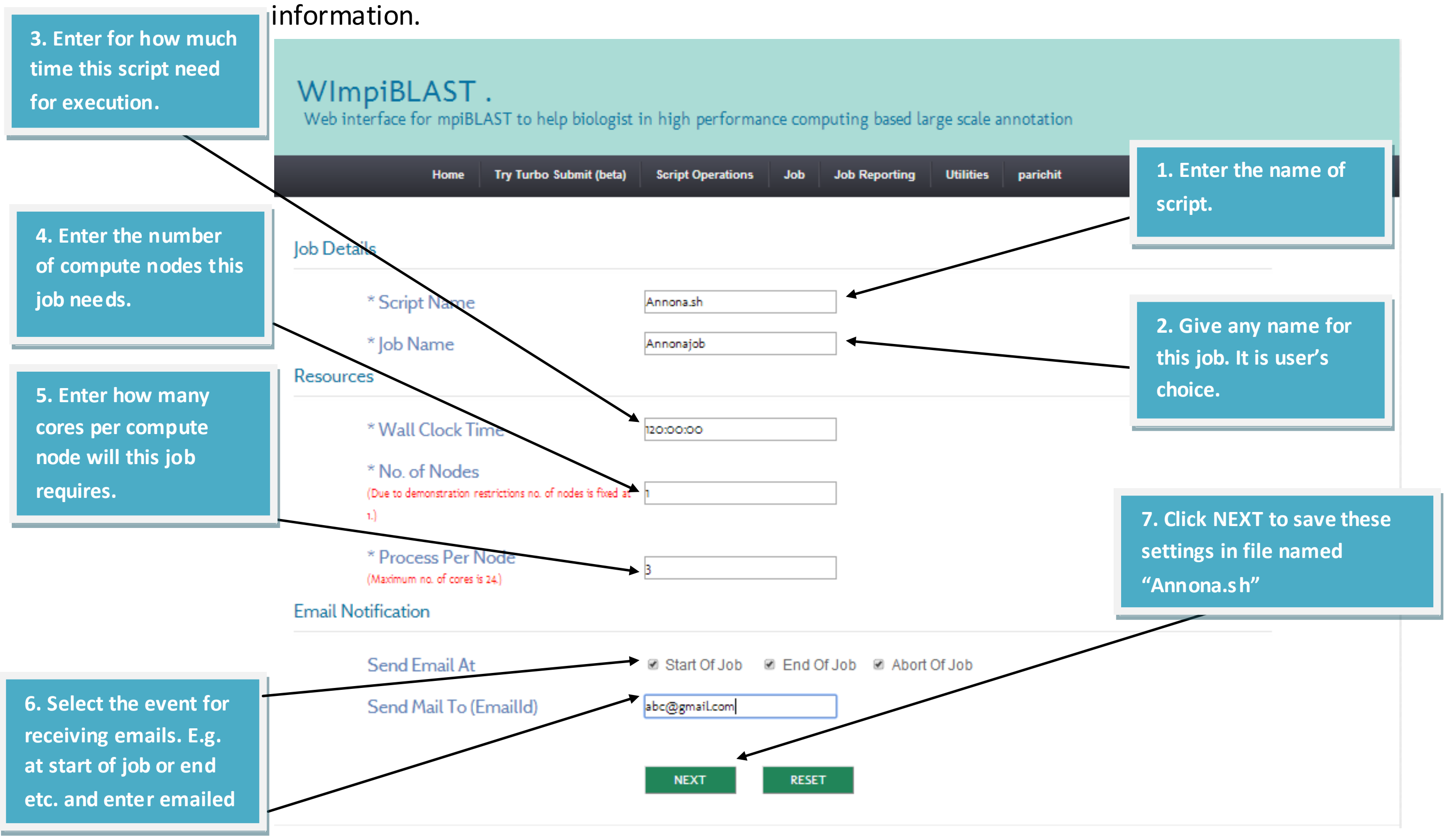

3. After clicking on "**NEXT**" button, user will be redirected to mpiBLAST specific input section. Enter required details.

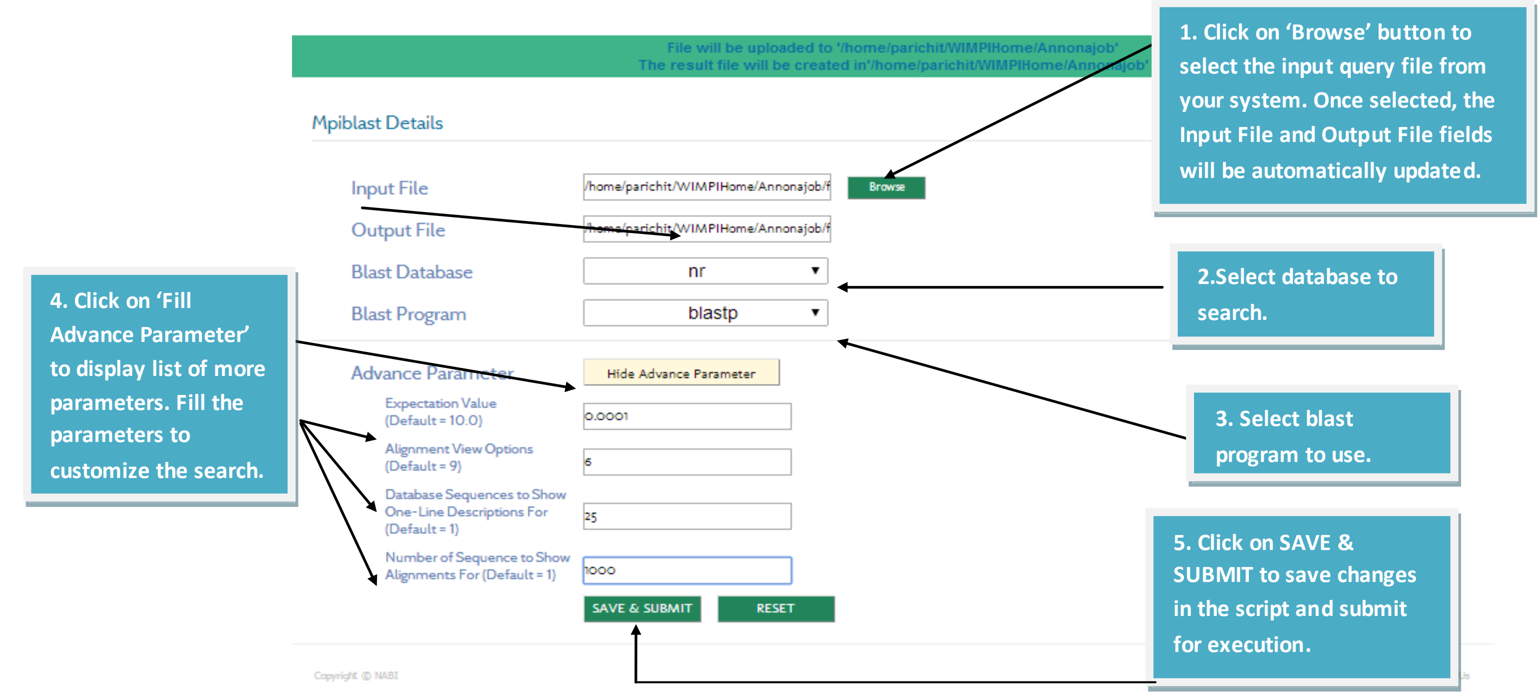

You have completed your first job submission through WImpiBLAST.

How to change previous scripts: This section explains how to change previously created scripts.

1. Select "**Modify Older Script**" option from Script operations tab.

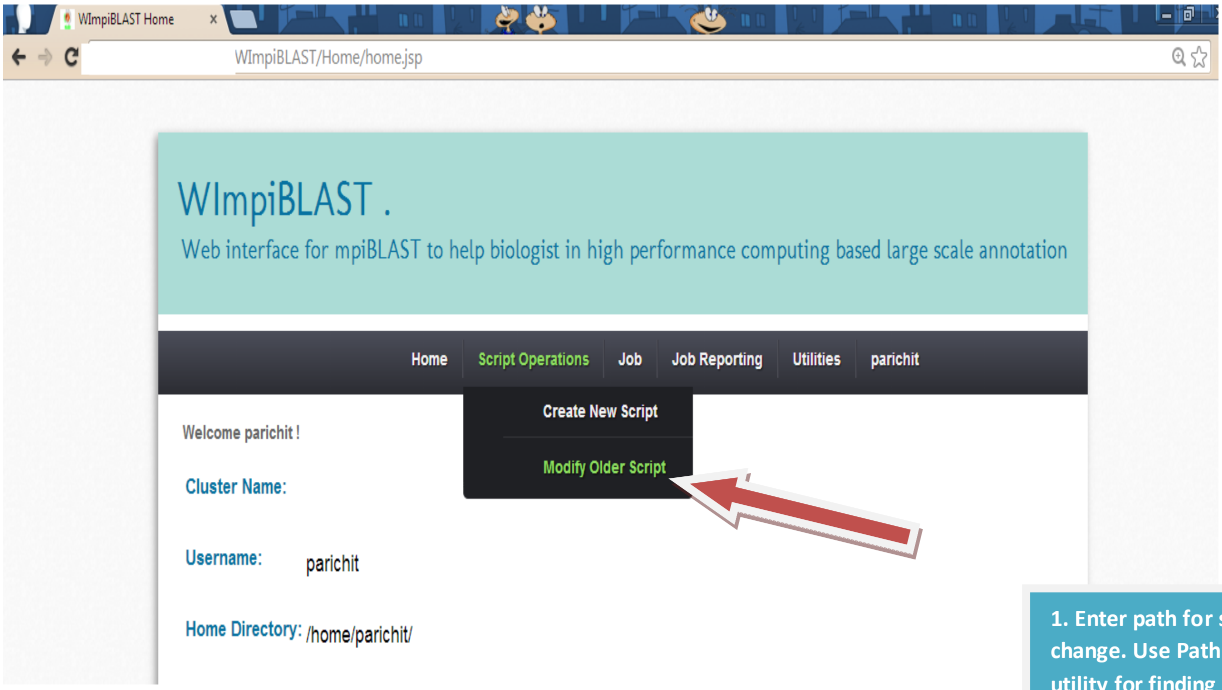

2. After clicking on Modify older Script following page will be opened.

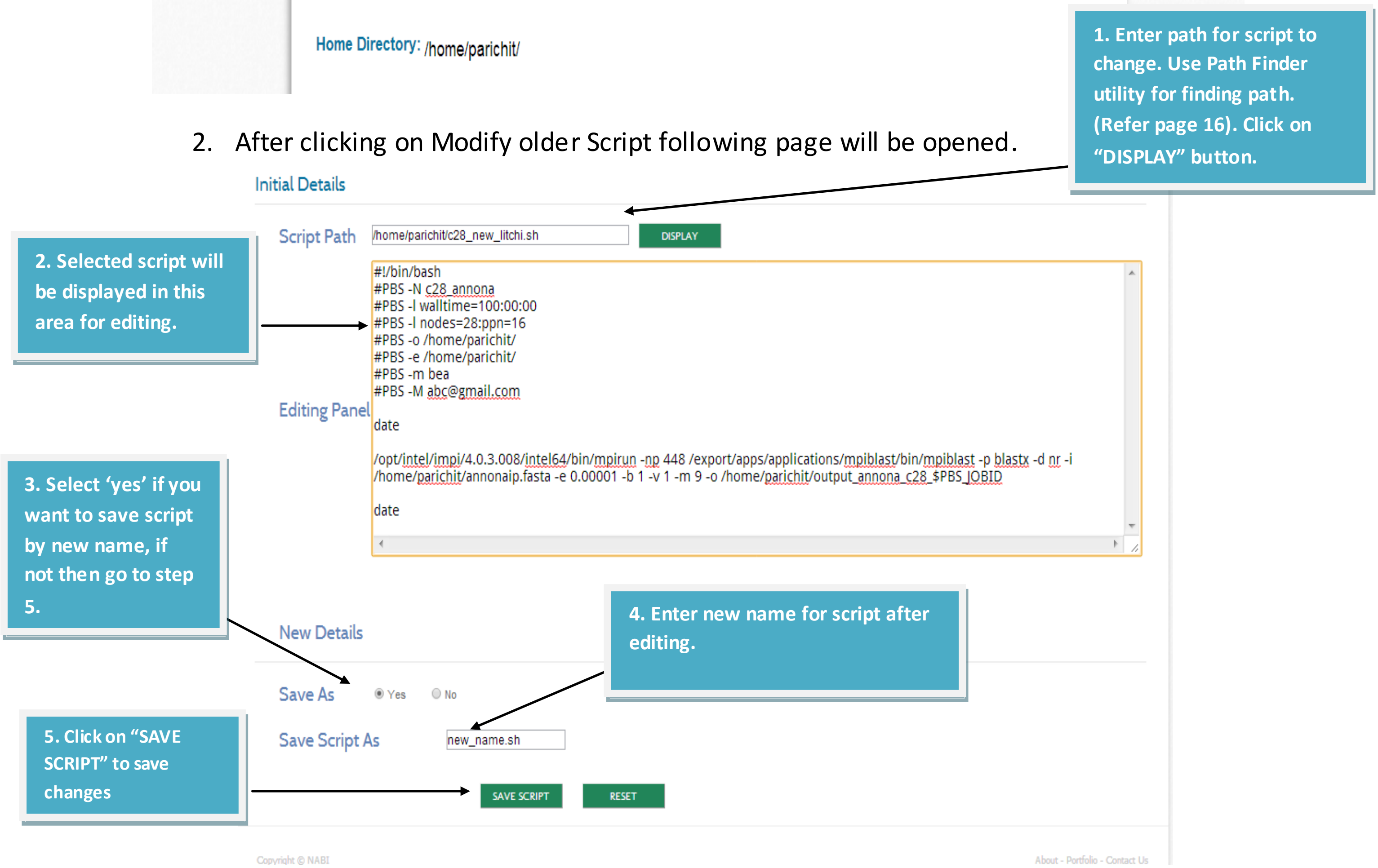

You have completed how to change old Script.

How to submit script after modifying: Sometime you may need to just submit the script after making some changes rather than following whole process of 'Create & Submit Script'. To just submit the script, follow these steps,

1. Go to '**Submit Job option**' in job tab.

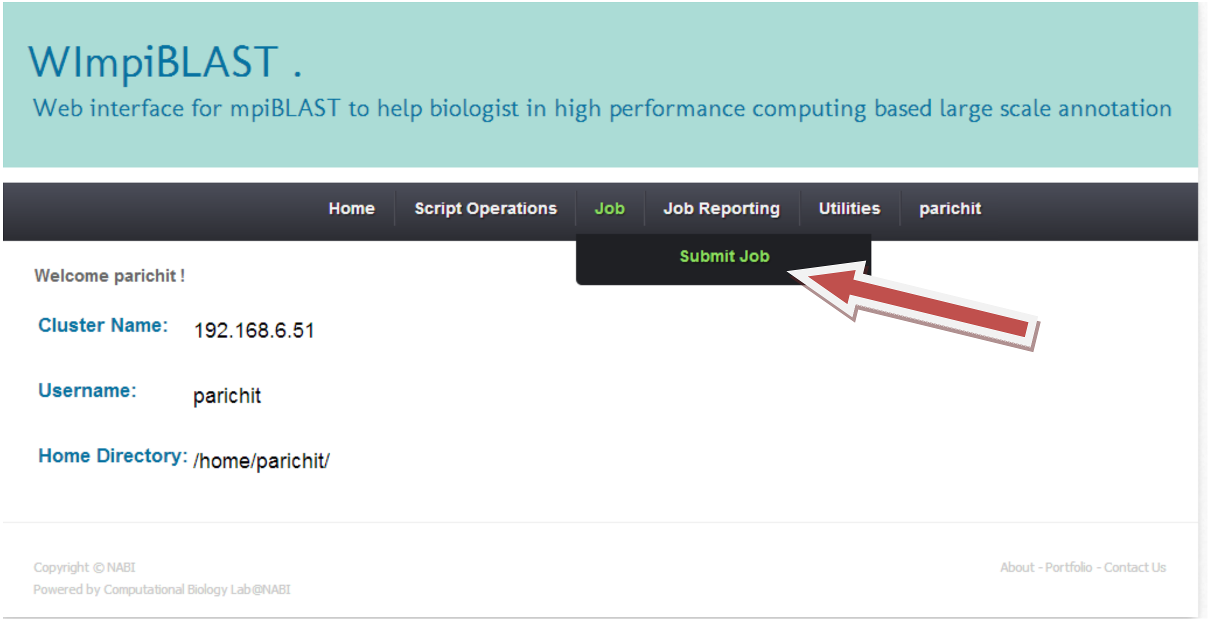

2. After clicking on **Submit Job** following page will be opened, enter required details.

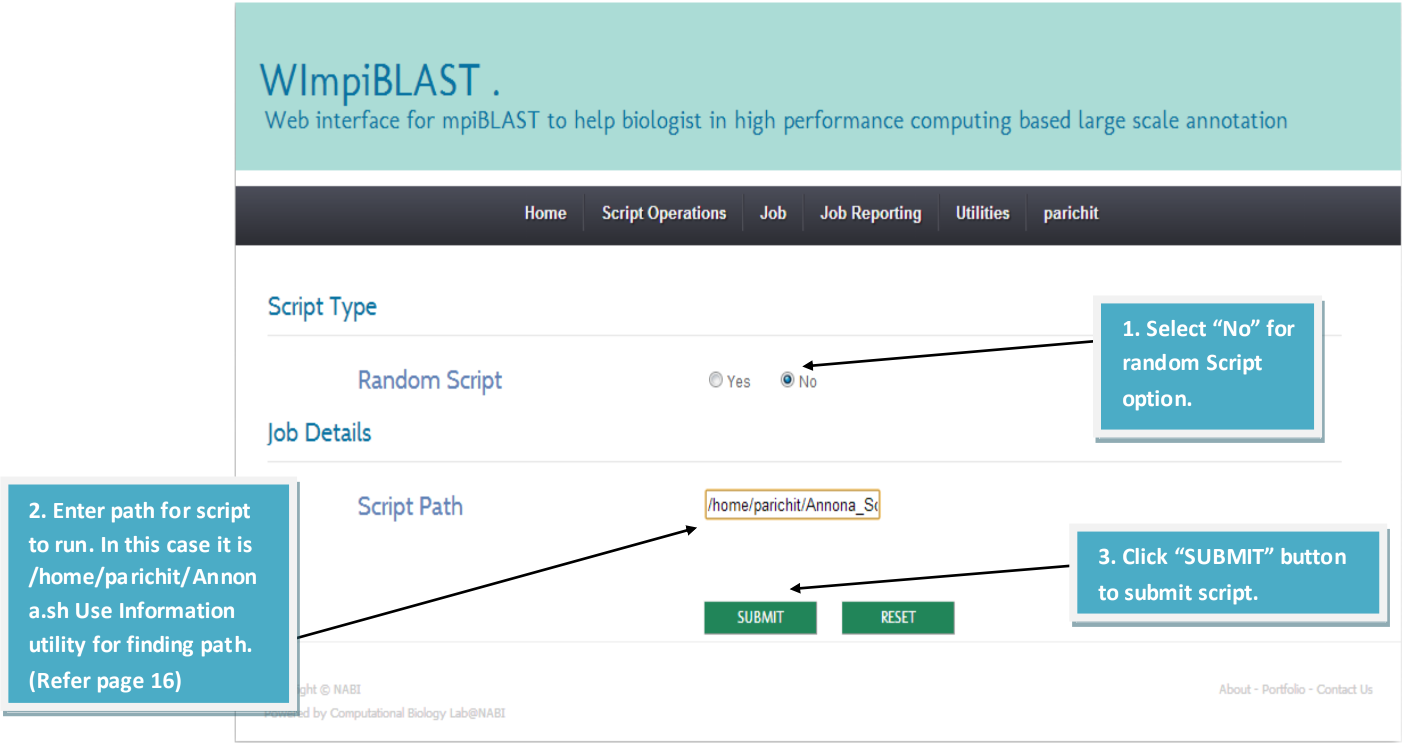

You have completed how to submit previous script after modifying.

How to monitor running scripts: This section explains how to keep track of jobs submitted jobs. To see running scripts or jobs in WImpiBLAST follow these steps,

Job: In the context of this document a job is a script that has been submitted for execution.

1. Go to Job Reporting tab and select **View Jobs** option as highlighted in following picture.

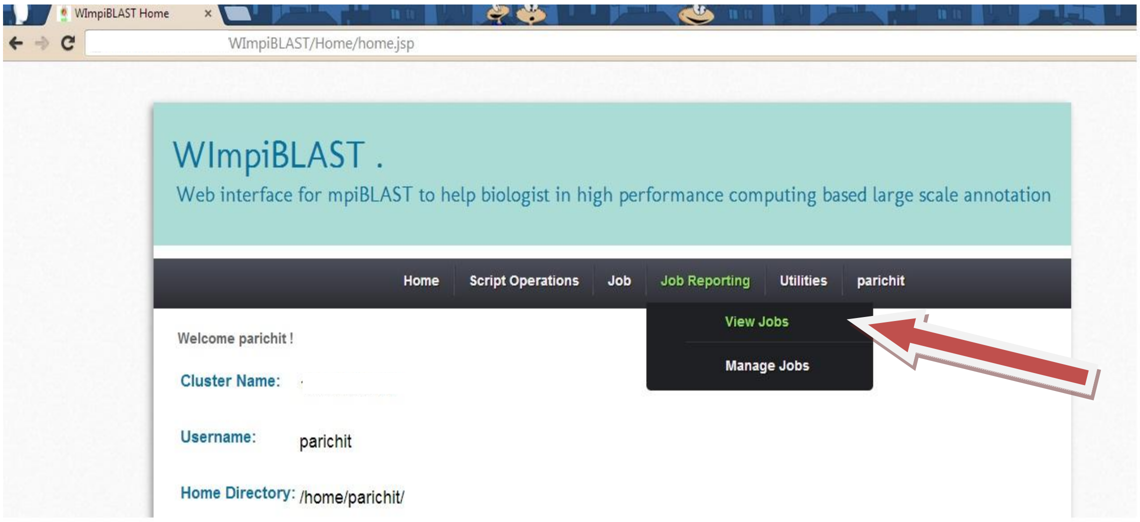

2. After clicking on '**View Jobs'** following page will show all jobs submitted by user.

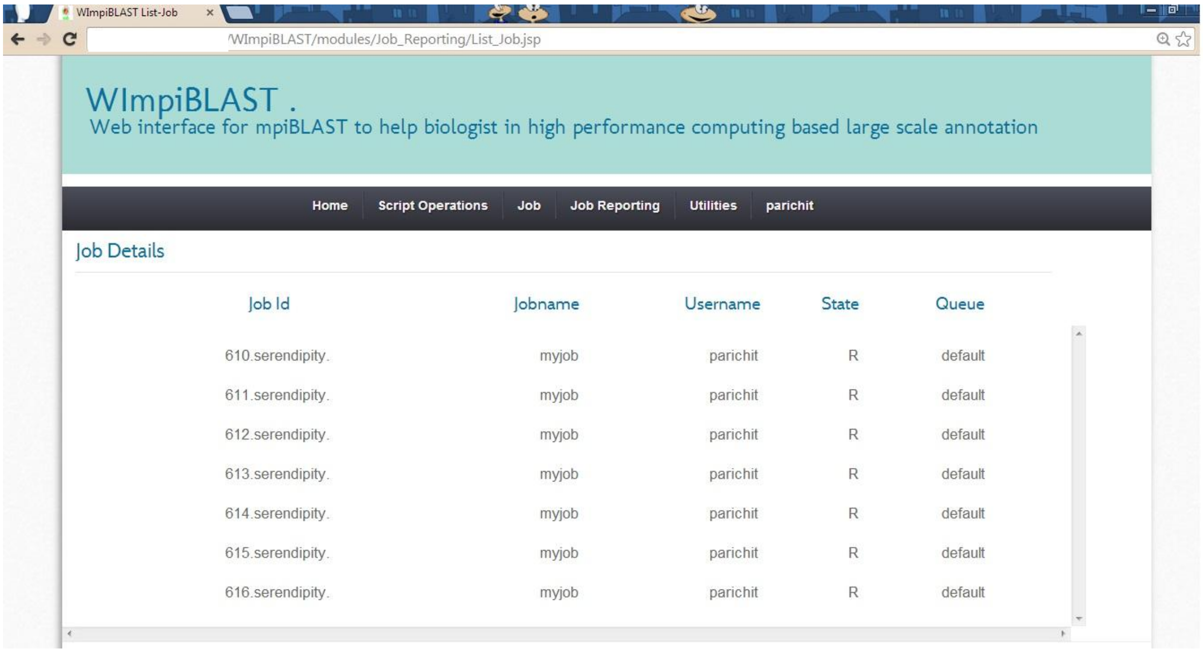

You have completed How to View Job

How to manage running scripts: This section explains how to manage submitted jobs. To manage jobs in WImpiBLAST follow these steps,

Job: In the context of this document a job is a script that has been submitted for execution.

1. Go to Job Reporting tab and select '**Manage Jobs**' option as highlighted in following picture.

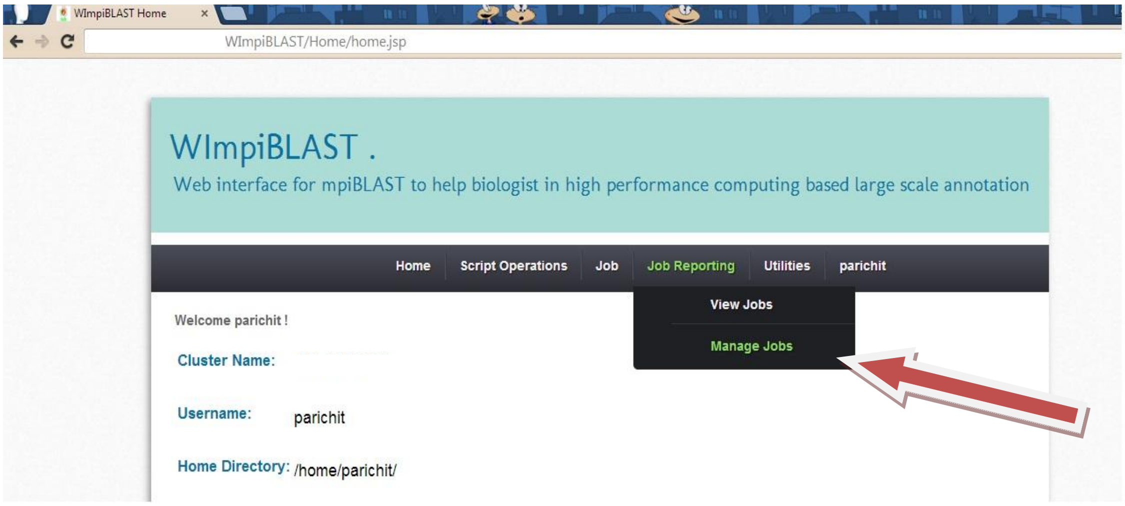

2. After clicking on '**Manage Jobs'** following page will open that will allow user to '**Delete**', '**Hole**' and '**Release**' running jobs.

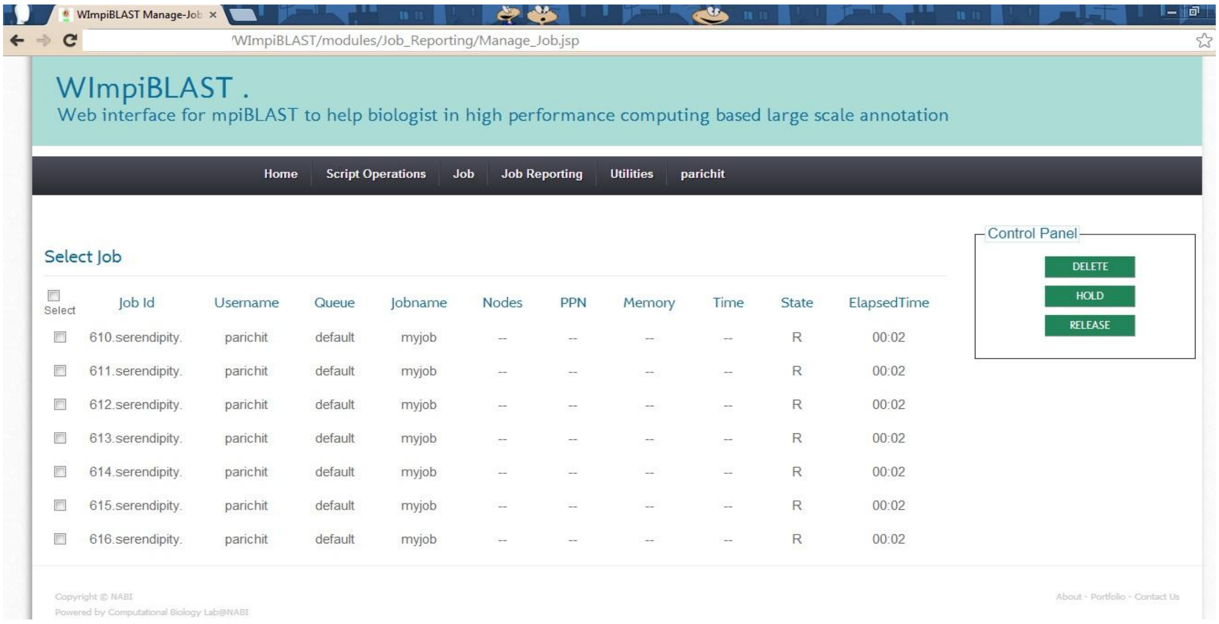

3. Select any job by checking the box on left most side job (marked by orange arrow) and click on buttons to perform action (marked by red arrow). For e.g. after checking box of any job click on '**DELETE**' button on right side to delete that job from system.

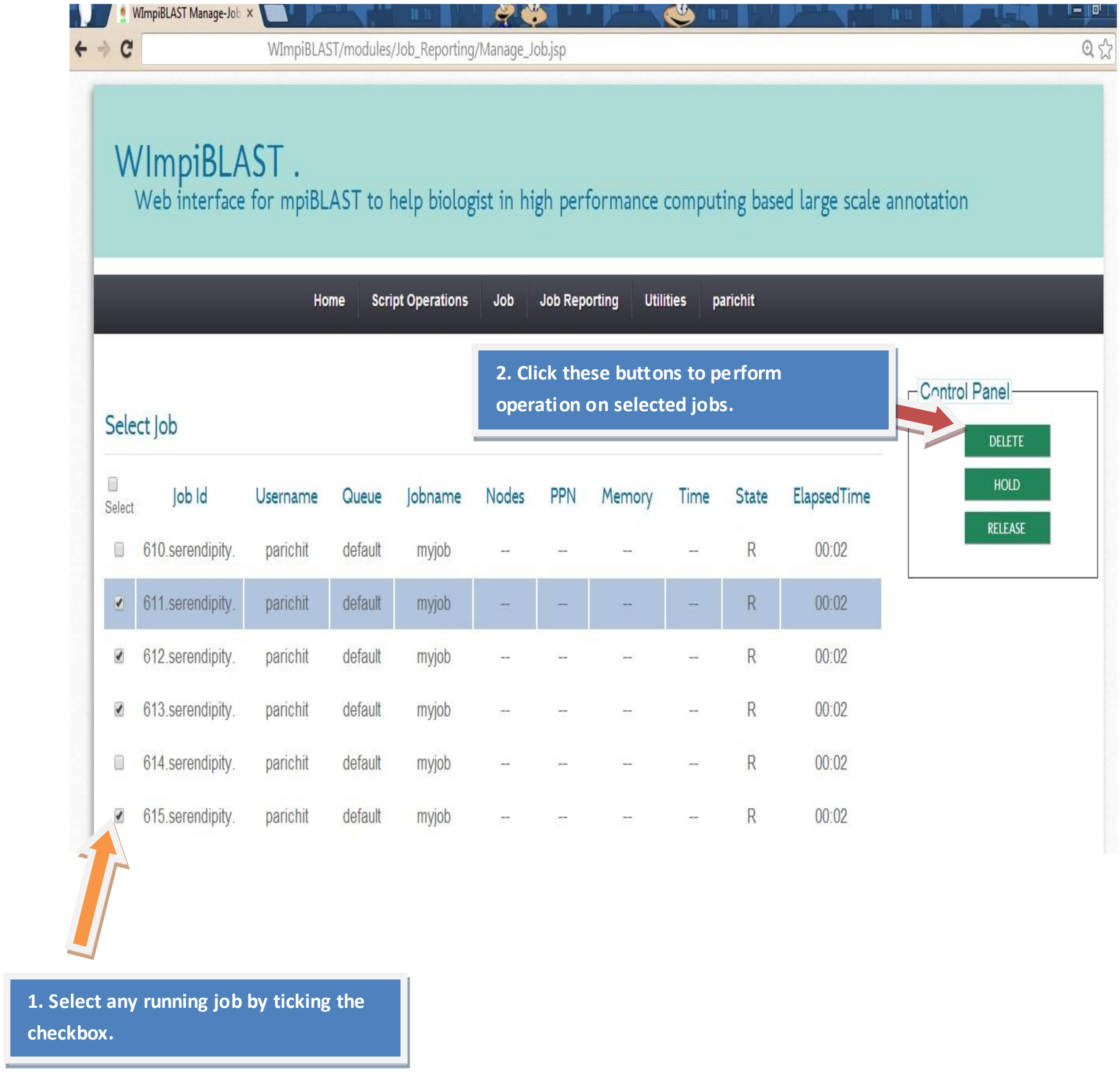

You have completed How to Manage Job

How to manage files in WImpiBLAST: This section describes how user can download their files; upload their query files, view files online and do selective file display in WImpiBLAST.

1. Go to Utilities tab from navigation bar and select '**File Manager**' as marked by red arrow in following picture.

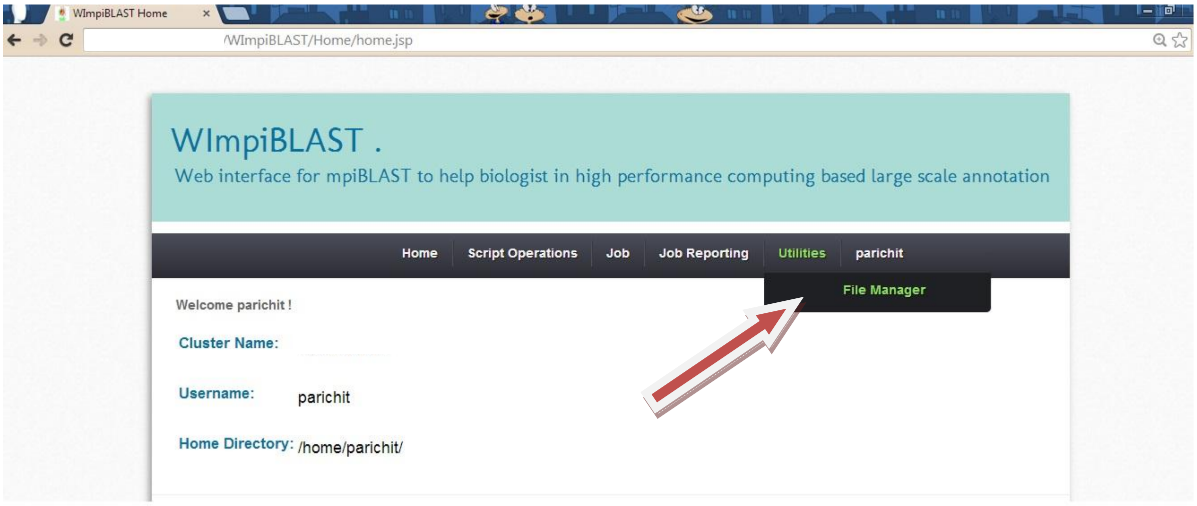

2. After clicking on '**File Manager'** following page will open. Click on '**Tree View'** button on upper left corner of page as shown by red arrow in following picture.

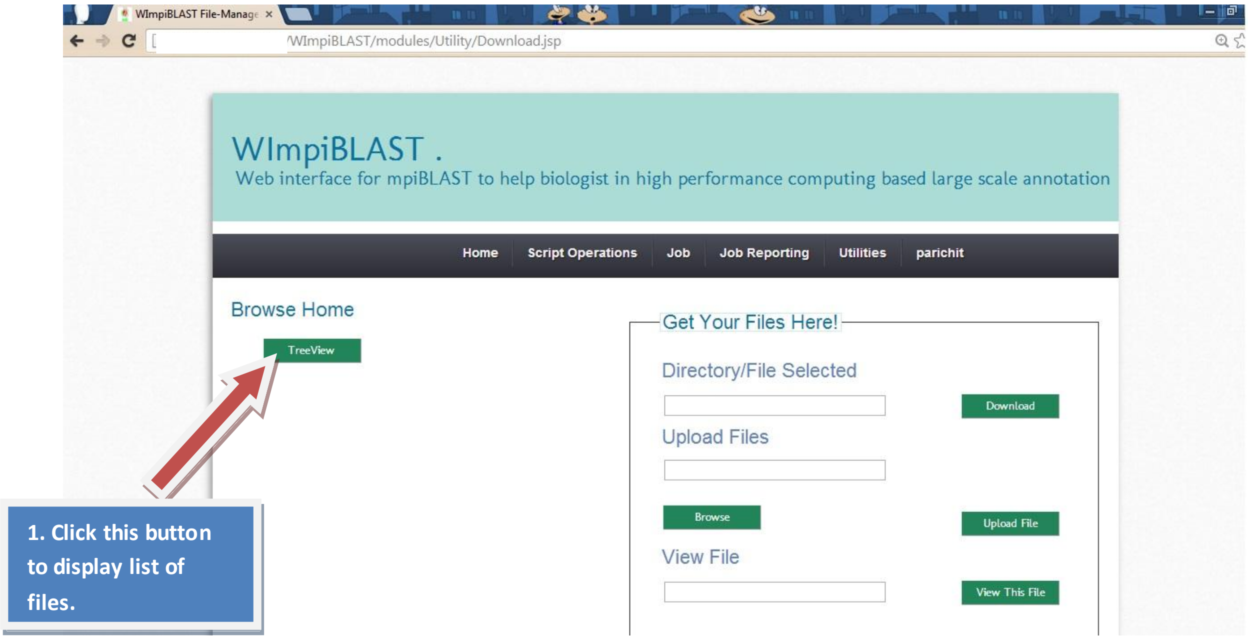

3. After clicking on **Tree View** the contents of your home directory will be displayed as shown in following figure (red arrow). All your files and folder can be seen inside this list.

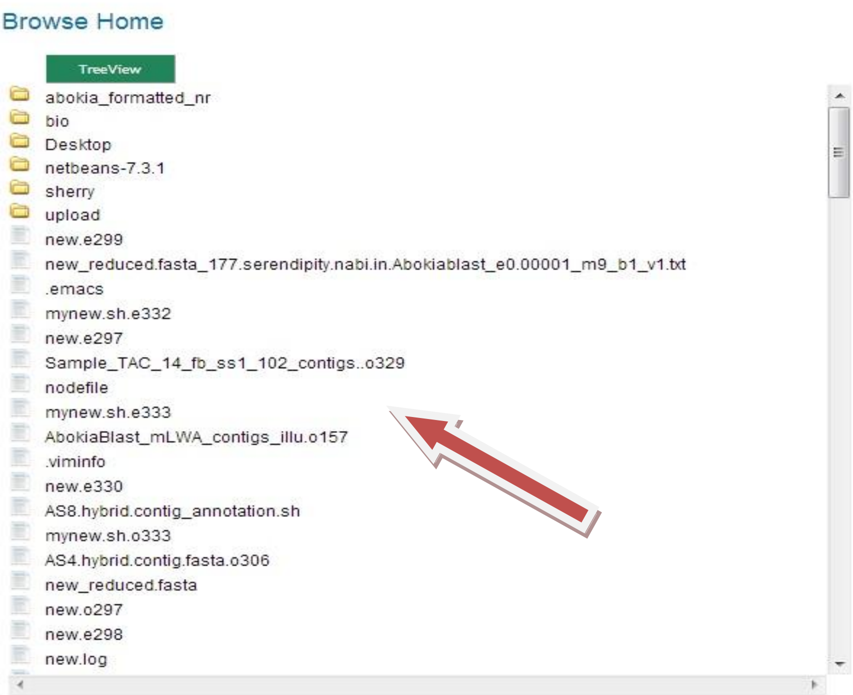

(a) To download file: Select any file from displayed list (refer to point 'a' to display a list of files), once you have selected file its name will displayed under 'Directory/File Selected' field. To start download click on **Download** button in right side of page.

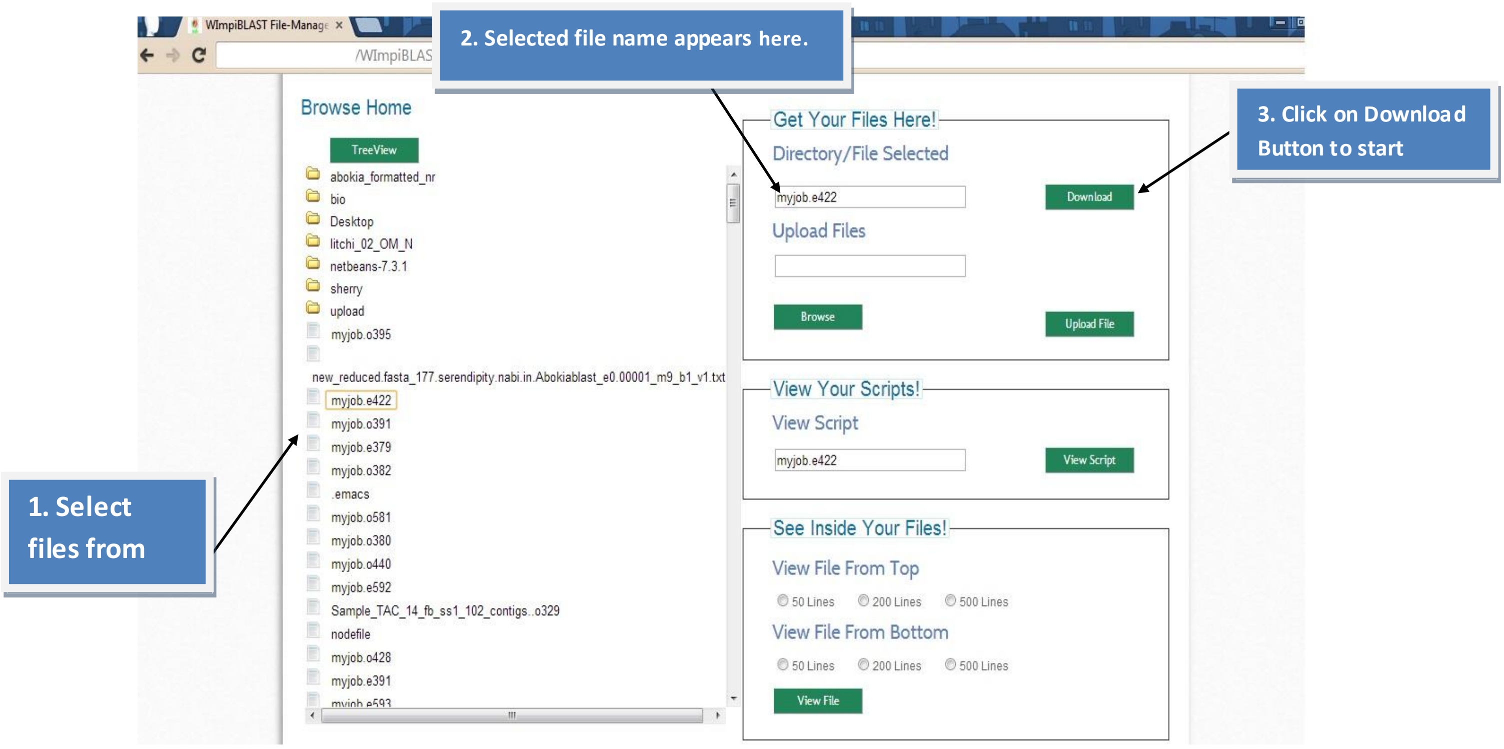

You have completed How to Download File.

(b) To Upload file: Click on '**Browse**' (marked by red arrow) button to select any file from your system (marked by orange arrow) that you want to upload.

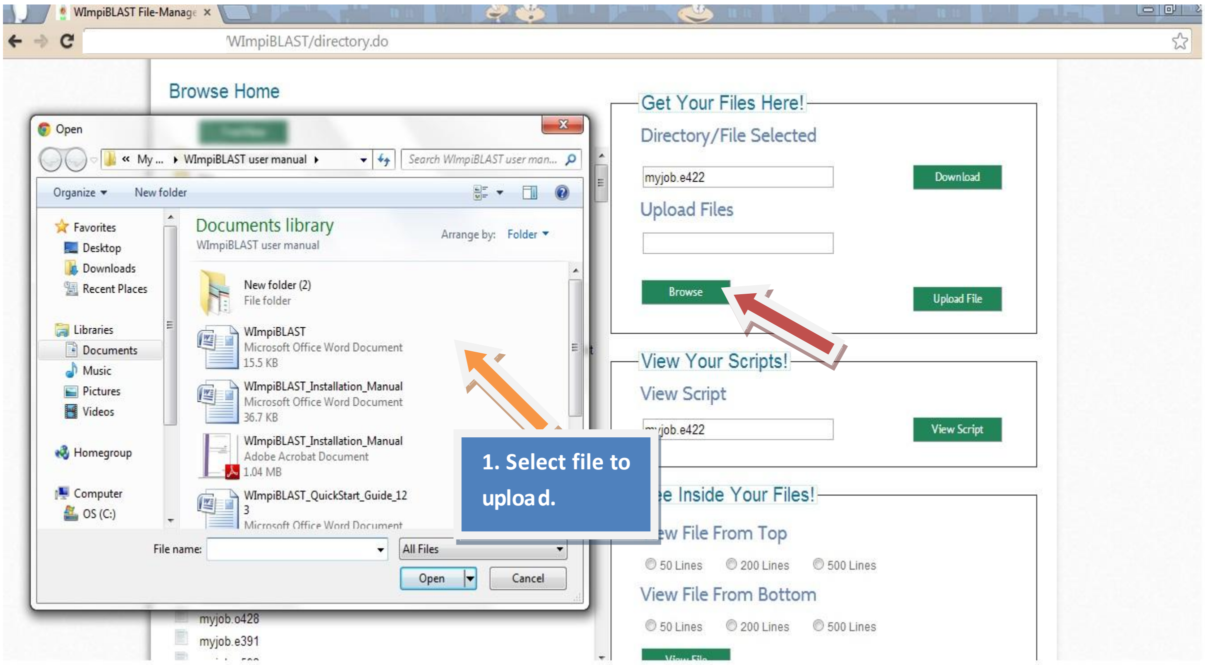

(c) Once you have selected the file its name will display under 'Upload Files Field' (marked by orange arrow). To start uploading press '**Upload File'** button (marked by red arrow).

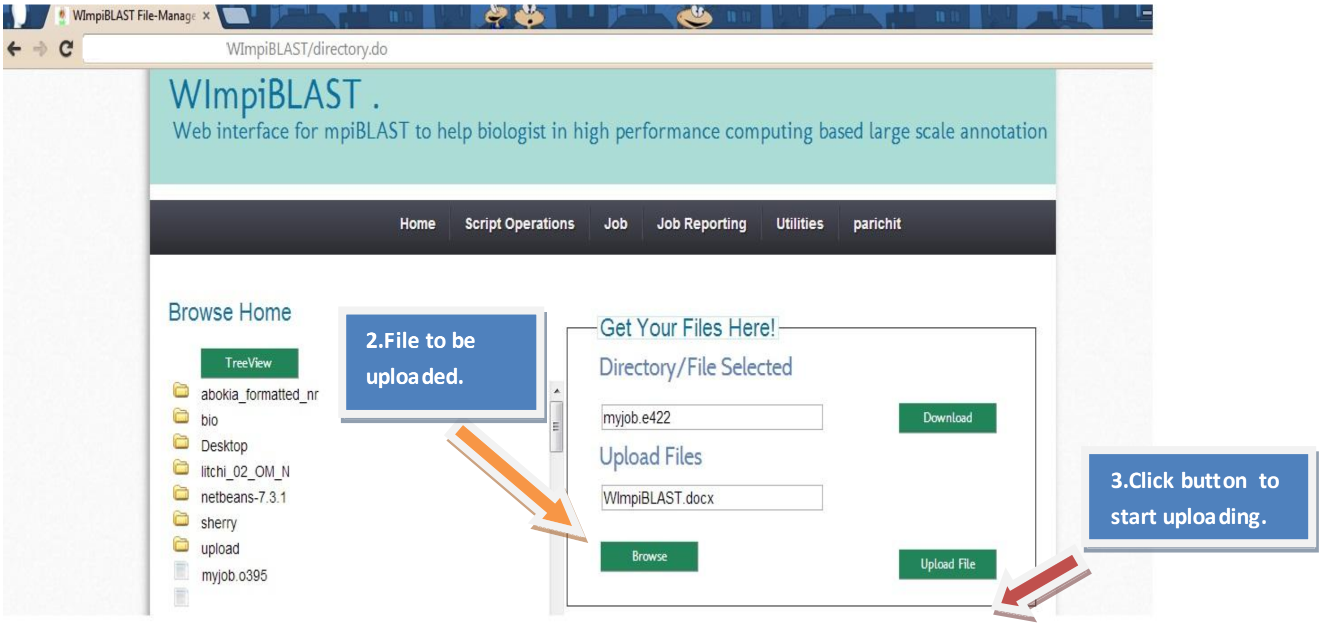

You have completed How to Upload file.

(d) To view script: To view any script in browser window, select any script from displayed list of files (refer to pint 'a' to display a list of files). Once selected its name will appear under 'View Script' field (orange arrow). To view script click on '**View Script'** button (red arrow).

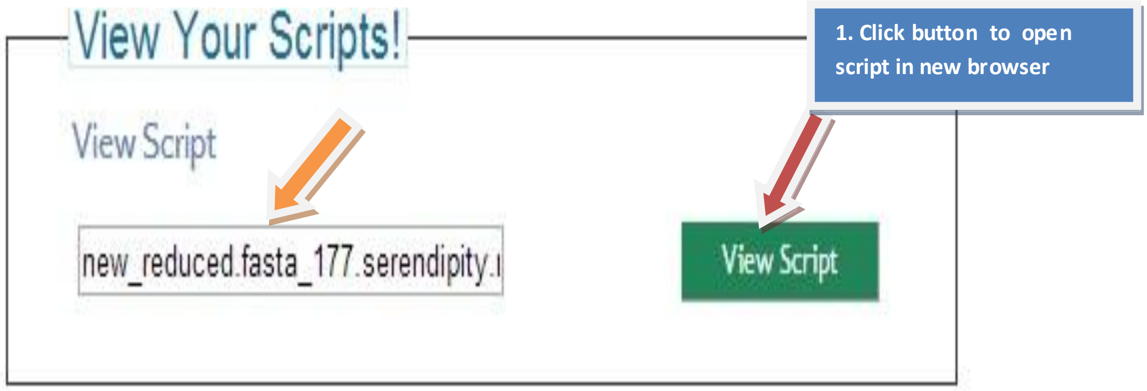

(e) To see selective part of files when files are large: For files greater than 2 MB View Script will not work. Large files can only be opened by selective display feature that displays only selected part of file in browser i.e. 500 line only, 200 line only etc.
To view selective part in browser window, select any file from displayed list of files (refer to pint 'a' to display a list of files). Choose number of lines to be displayed e.g. **50 lines from top or 500 lines from bottom** (orange arrow). To view file click on **'View File'** button (red arrow).

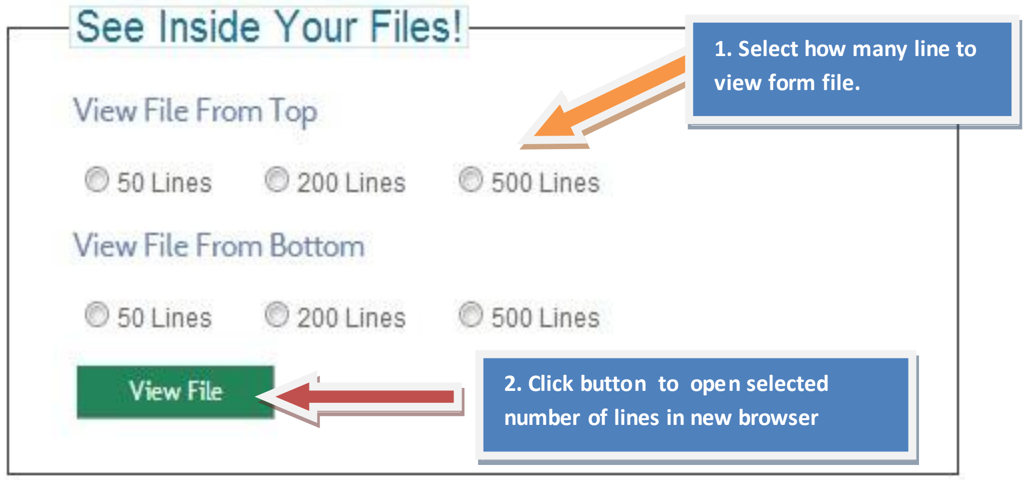

You have completed View Script and Selective View.

(f) How to use Path Information Utility: At several places you will need to provide paths for creating script, path for script to be modified or path to submit job. Follow below steps to generate paths using Path Finder utility

Follow step 2 (page number 12) to generate contents of home directory.

Click on any file or directory in the list generated by Tree View (orange arrow), Corresponding path of that file or directory will be generated in text fields as shown below (red arrow).

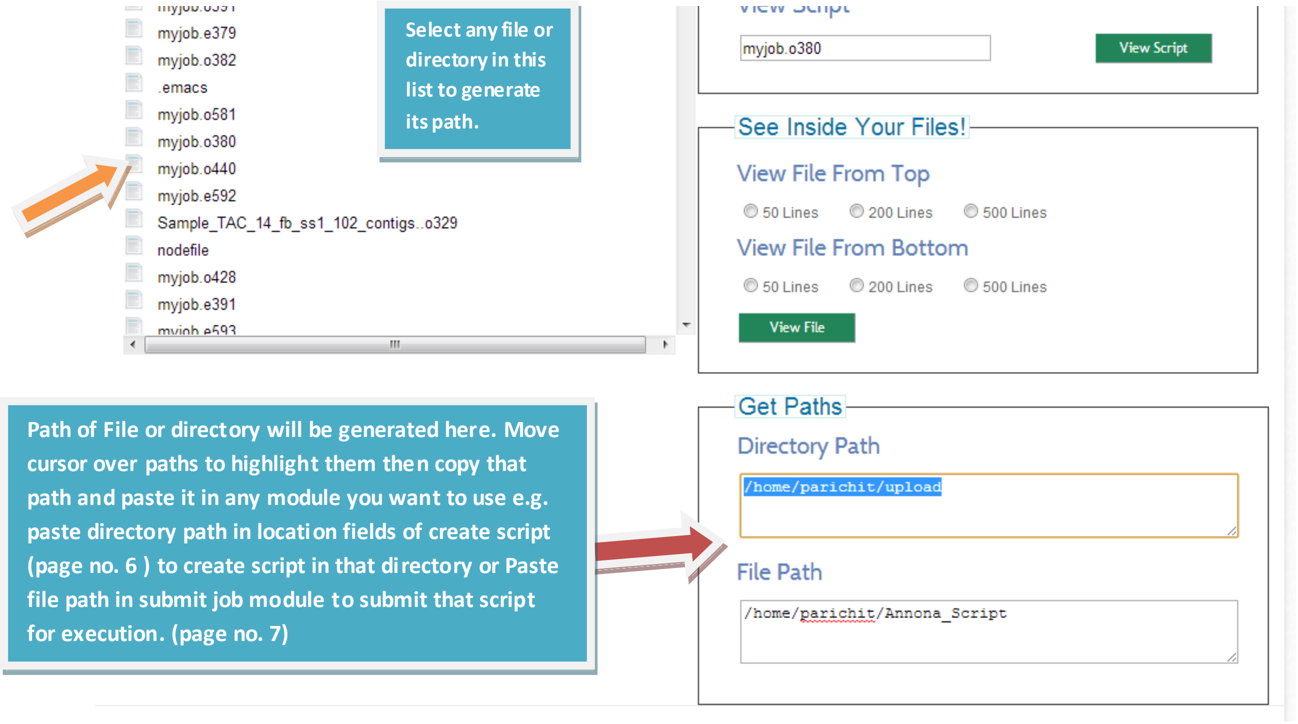

You have completed how to use Path Information Utility

How to Logout: It is very important from safety and data confidentiality point of view to logout of portal after completing activity. To logout from portal follow these steps:

Click on **Logout** option from **username** fields on navigation menu. (red arrow)

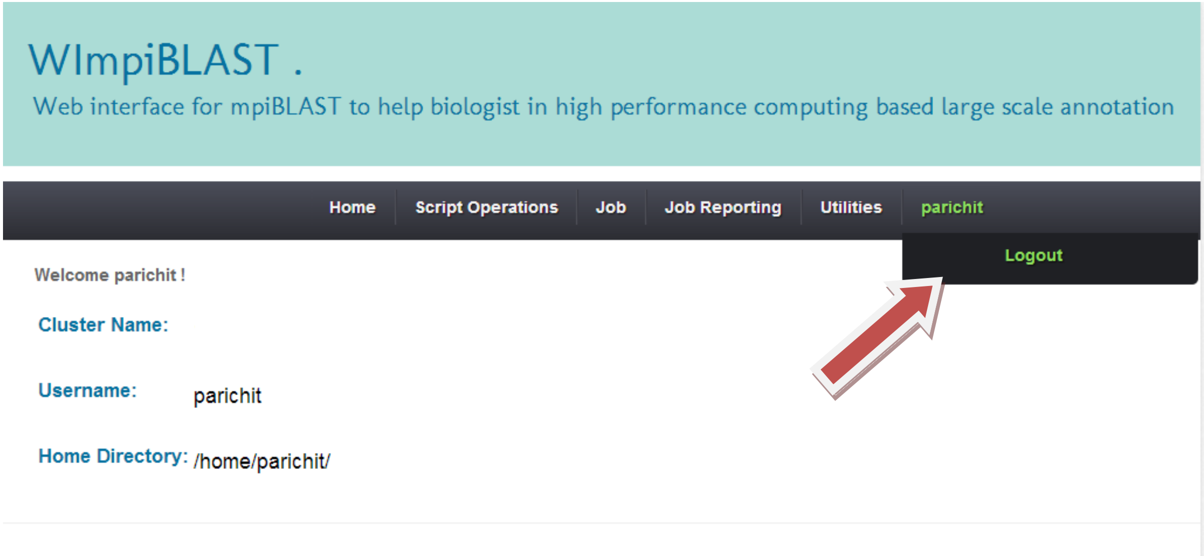

You have completed how to logout.

Note: For help on how to set up or install WImpiBLAST please refer to Installation manual.

Appendix

1. **Deployment Server or Hosting Server:** This term refers to system on which WImpiBLAST.war file is copied and all the users will access WImpiBLAST by using ipaddress of this server in their browser.

2. **mpirun/mpiexec path:** Administrator can provide either mpirun path or mpiexec path whichever is present on their system.

   For details see: http://www.mpich.org/

3. **User Accessible**: mpirun, mpiexec and mpiblast binaries should be accessible by all the users of the system i.e these binaries should be present at path that is readable and executable by all users but writable by only administrator or root.

4. **Mpi:** Message Passing Interface is a library or collection of routines and functions to enable distributed communication and i/o in multi-core systems or HPC clusters or Supercomputers.

   For details: https://computing.llnl.gov/tutorials/mpi/

5. **HPC:** High Performance Computing refers to powerful computing systems that can used to speed up otherwise intensive calculations.

6. **BLAST:** Basic Local Alignment Search Tool is a Sequence Similarity Search application used for annotation of genome.

   For Details: http://blast.ncbi.nlm.nih.gov/Blast.cgi

7. **Job:** In context of this document only, a job refers to a executable script that is submitted for execution to job scheduler by user.

8. **mpiBLAST:** mpiBLAST is parallel version of NCBI's BLAST application.

   For Details: www.mpiblast.org

Note: Dear user, we have tried our best to help administrator and users in understanding how to use WImpiBLAST but if you think there need be improvements or if you face bugs please feel free to report any issue or bug at www.ict@nabi.res.in or use https://groups.google.com/forum/#!forum/wimpiblast-user-group to ask your questions.

We are a small team of computational biologists and computer science guys who sincerely want to contribute to field of computational biology by helping you out. In case we omitted something or do not incorporate some critical feature that you think can make difference then kindly let us know.

We will try our best to sort you out.
