# Supplementary material for: WImpiBLAST: Web Interface for mpiBLAST to Help Biologists Perform Large-Scale Annotation Using High Performance Computing
Source: PLoS One. 2014 Jun 30;9(6):e101144. doi: 10.1371/journal.pone.0101144 (PMC4076281; doi:10.1371/journal.pone.0101144)
Supplement: Text S4 — Hyperlinks to fasta file containing sequences used in use case runs. (DOCX) [file pone.0101144.s006.docx]

**Text S4: Hyperlinks to fasta file containing sequences used in use case runs .**

http://wimpiblast.nabi.res.in/WImpiBLAST/gen/ss1_Table_4_and_5_Sequences.fasta.zip

http://wimpiblast.nabi.res.in/WImpiBLAST/gen/ss2_Table_6_Sequences.fasta.zip

http://wimpiblast.nabi.res.in/WImpiBLAST/gen/ss3_Table_7_Sequences.fasta.zip
